# Cathelicidin antimicrobial peptide expression in neutrophils and neurons antagonistically modulates neuroinflammation

**DOI:** 10.1172/JCI184502

**Published:** 2024-12-10

**Authors:** Subash Chand Verma, Emmanuelle Enée, Kanchanadevi Manasse, Feriel Rebhi, Axelle Penc, David Romeo-Guitart, Cuc Bui Thi, Matthias Titeux, Franck Oury, Simon Fillatreau, Roland Liblau, Julien Diana

**Affiliations:** 1 Université Paris Cité, CNRS, INSERM, Institut Necker Enfants Malades–INEM, Paris, France.; 2 Université Paris Cité, Imagine Institute, INSERM U1163, Paris, France.; 3 APHP, Hôpital Necker-Enfants Malades, Paris, France.; 4 Toulouse Institute for Infectious and Inflammatory Diseases (Infinity), CNRS, INSERM, Université Paul-Sabatier de Toulouse (UPS), Toulouse, France.; 5 Department of Immunology, Toulouse University Hospital, Toulouse, France.

**Keywords:** Autoimmunity, Immunology, Innate immunity, Multiple sclerosis, Neutrophils

## Abstract

Multiple sclerosis (MS) is an autoimmune disease that affects the CNS, the pathophysiology of which remains unclear and for which there is no definitive cure. Antimicrobial peptides (AMPs) are immunomodulatory molecules expressed in various tissues, including the CNS. Here, we investigated whether the cathelicidin-related AMP (CRAMP) modulated the development of experimental autoimmune encephalomyelitis (EAE), a mouse model of MS. We showed that, at an early stage, CNS-recruited neutrophils produced neutrophil extracellular traps (NETs) rich in CRAMP that were required for EAE initiation. NET-associated CRAMP stimulated IL-6 production by dendritic cells via the cGAS/STING pathway, thereby promoting encephalitogenic Th17 response. However, at a later disease stage, neurons also expressed CRAMP that reduced EAE severity. *Camp* knockdown in neurons led to disease exacerbation, while local injection of CRAMP_1–39_ at the peak of EAE promoted disease remission. In vitro, CRAMP_1–39_ regulated the activation of microglia and astrocytes through the formyl peptide receptor (FPR) 2. Finally, administration of butyrate, a gut microbiota-derived metabolite, stimulated the expression of neural CRAMP via the free fatty acids receptors 2/3 (FFAR2/3), and prevented EAE. This study shows that CRAMP produced by different cell types has opposing effects on neuroinflammation, offering therapeutic opportunities for MS and other neuroinflammatory disorders.

## Abstract 2

The antimicrobial peptide cathelicidin plays opposing roles depending on its cellular source in neuroinflammation in a preclinical model of multiple sclerosis.

## Graphical abstract

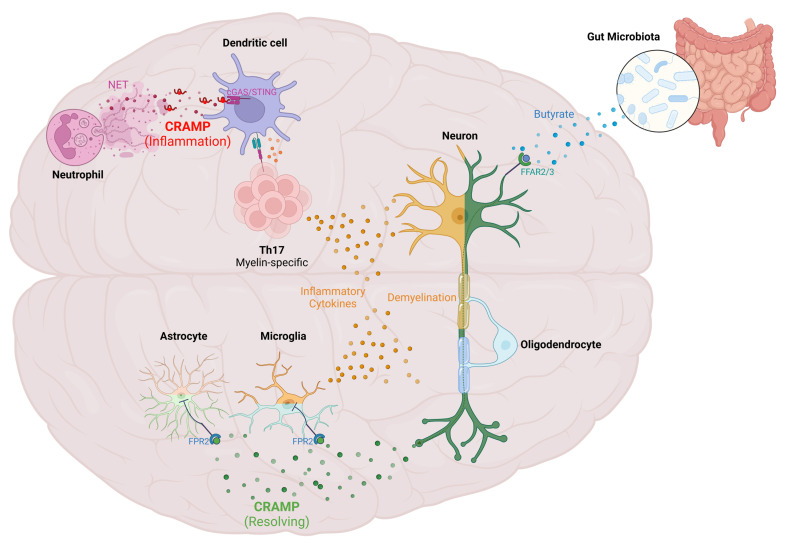

## Introduction

Multiple sclerosis (MS) is an autoimmune disease that affects the CNS and exhibits increasing incidence. The hallmarks of MS include CNS infiltration by immune cells, activation of glial cells, multifocal demyelination, and axonal loss. The disease is influenced by multiple genetic polymorphisms and environmental factors including the intestinal microbiota, which is thought to play a critical role in the development of the disease (1, 2). There is still no satisfying therapy for MS (3).

A better understanding of the immune processes at play in MS has been achieved using the preclinical model experimental autoimmune encephalomyelitis (EAE) (4). EAE can be induced by immunization with various myelin antigens such as myelin oligodendrocyte glycoprotein (MOG) activating self-reactive CD4^+^ Th1 and 17 cells. Upon migration to the CNS, T cells initiate a local inflammatory response leading to axonal demyelination (5). Innate immune cells such as microglia, dendritic cells, and macrophages play a critical role in this local inflammatory response (6, 7).

The relevance of antimicrobial peptides (AMPs) in autoimmunity is increasingly acknowledged. AMPs were discovered more than 40 years ago and represent a crucial component of the innate immune system in all living organisms (8). They are mainly produced at epithelial surfaces and protect against invading microorganisms while maintaining the homeostasis of the commensal microbiota (9, 10). AMPs also have important immunomodulatory roles and can be either pro- or antiinflammatory depending on the context (11). For instance, AMPs play a role in the immunopathogenesis of various autoimmune diseases. AMPs from activated neutrophils can bind various TLR ligands and activate the immune system, contributing to the initiation of autoimmunity (12). A mechanism common to many autoimmune and autoinflammatory diseases is the ability of cathelicidin from neutrophil extracellular traps (NETs), complexed with nucleic acids, to induce inflammatory cytokines from myeloid cells (13–15). In contrast, AMPs secreted by nonimmune cells can prevent autoimmune responses by inducing various regulatory immune cell types, including regulatory macrophages and B cells, by engaging their receptors at the surface of these cells (16).

Emerging evidence suggests that AMPs are a part of the immune defense of the CNS (17, 18). AMPs are expressed in the CNS of rodents and humans at a steady state, and their expression increases in the context of infection (19–23). Following bacterial infection, cathelicidin-related AMP (CRAMP) is expressed in the CNS by recruited neutrophils, as well as by activated astrocytes, microglia, and meningeal cells (21, 24). In an experimental model of pneumococcal meningitis, mice deficient in CRAMP showed a higher degree of glial cell activation and CNS inflammation (25, 26).

In this study, we investigated whether CRAMP is expressed in the CNS during the development of EAE and whether it plays a critical role in controlling neuroinflammation.

## Results

### CRAMP shows opposite roles during EAE.

**Figure 1:**
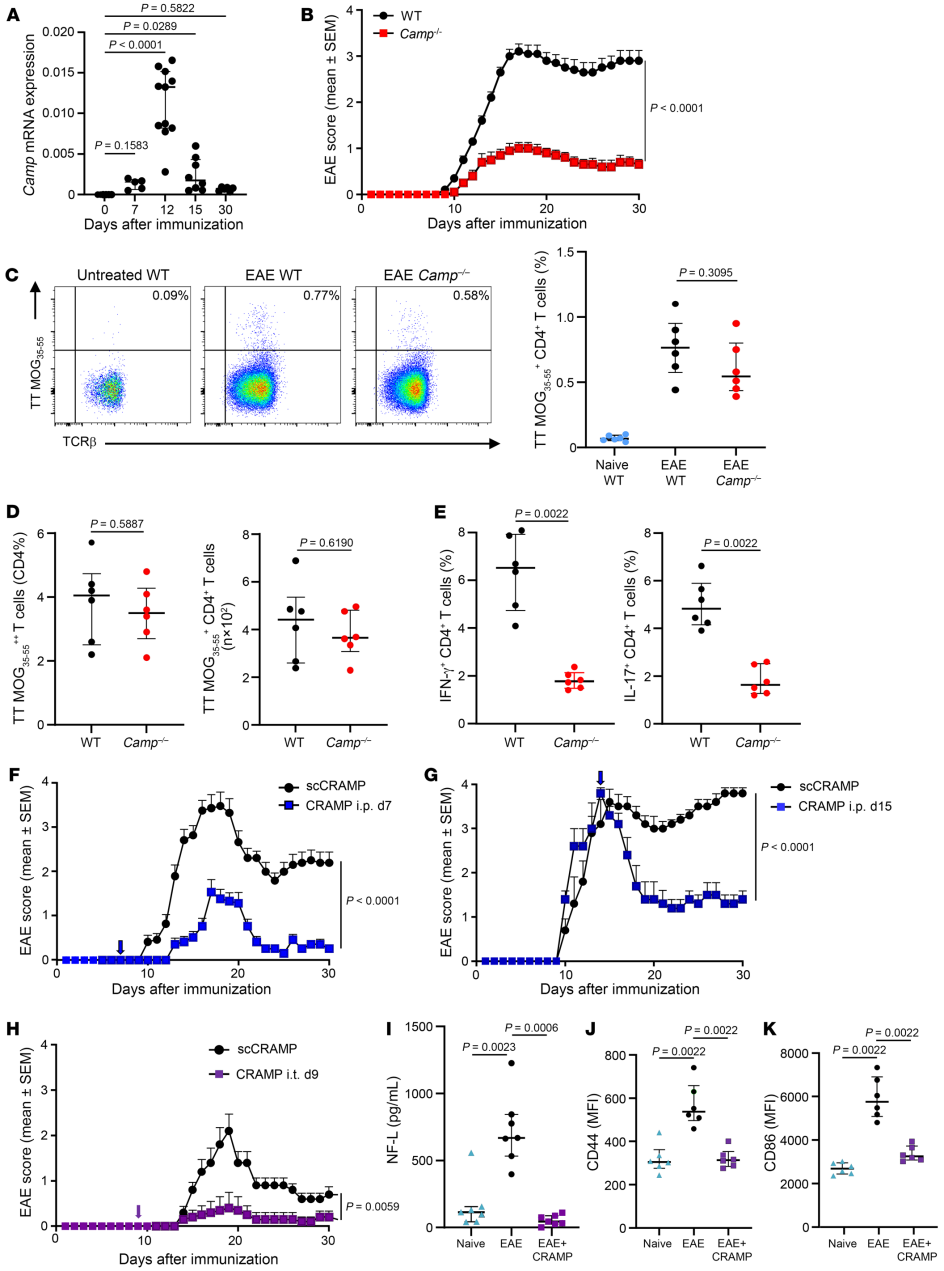
Opposite role of CRAMP during EAE. (**A**) mRNA expression of CRAMP was analyzed by RT-qPCR in SC from C57BL/6 WT mice at different days after EAE induction. Data are the median plus or minus interquartile range of 5 to 11 independent mice per group from 4 independent experiments. (**B**) *Camp*^–/–^ C57BL/6 mice and WT littermate controls (*n* = 30 mice per group from 6 independent experiments) were immunized with MOG_35-55_ to induce EAE. Clinical scores are shown (data are represented as mean ± SEM). (**C** and **D**) Cells from draining lymph nodes (**C**) or SC (**D**) were recovered on day 7 or 12, respectively, after EAE induction in *Camp*^–/–^ and WT C57BL/6 mice and stained with I-A^b^ MOG_35-55_ tetramer. Data are the frequency and number of tetramer^+^ cells among CD45^+^βTCR^+^CD4^+^ cells. Median plus or minus interquartile range of 6 independent mice per group from 3 independent experiments is shown. (**E**) Frequency of IFN-γ^+^ and IL-17^+^ cells in CD4^+^ T cells recovered farom the SC at day 12 after EAE induction in *Camp*^–/–^ and WT C57BL/6 mice and restimulated for 6 hours with PMA/ionomycin. Median plus or minus interquartile range of 6 independent mice per group is shown from 3 independent experiments. (**F**−**H**) WT mice (*n* = 15 mice per group from 3 independent experiments) were immunized with MOG_35-55_ to induce EAE. Mice were treated with CRAMP or scCRAMP i.p. at day 7 (**F**) or at day 15 (**G**) or i.t. at day 9 (**H**) after EAE induction. Clinical scores are shown (data are represented as mean ± SEM). (**I**) NF-L protein levels were measured in the serum 15 days after EAE induction in WT mice treated as in **G** and in unmanipulated (naive) WT mice. Data are the median plus or minus interquartile range of 7 independent mice per group from 3 independent experiments. (**J** and **K**) Expression of CD44 on astrocytes (**J**) and CD86 on microglia (**K**) was determined by flow cytometry at day 15 after EAE induction in the SC of WT mice treated as in **G** and in unmanipulated (naive) WT mice. Data are the median plus or minus interquartile range of 6 independent mice per group from 3 independent experiments. Comparison between each group was performed using the nonparametric Mann-Whitney *U* test or Kruskal-Wallis test followed up by Dunn’s post test when more than 2 groups were compared. For evaluating differences between EAE scores over time, a 2-way ANOVA with Tukey’s multiple-comparisons test was used.

As a starting point, we evaluated the expression of CRAMP in the CNS during the development of EAE in C57BL/6 WT mice. The mRNA expression of *Camp*, encoding for CRAMP, increased in spinal cord (SC) during the course of EAE, peaking 12 days after immunization (Figure 1A). We observed that CFA was sufficient to induce *Camp* mRNA expression in the CNS, suggesting that peripheral inflammation can trigger *Camp* expression in the CNS (Supplemental Figure 1; supplemental material available online with this article; https://doi.org/10.1172/JCI184502DS1). Importantly, CRAMP-deficient *Camp*^–/–^ C57BL/6 mice exhibited significantly reduced EAE scores (Figure 1B), indicating that the expression of CRAMP was necessary for EAE development. To determine whether the absence of CRAMP prevented the initiation of the encephalitogenic T cell response in the periphery, we analyzed MOG_35-55_-specific CD4^+^ T cells using MOG_35-55_/IAb tetramer staining. Seven days after immunization, the frequency of MOG_35-55_-specific CD4^+^ T cells in the draining lymph nodes (dLNs) was comparable between WT and *Camp*^–/–^ mice, indicating that CRAMP did not play a role in the priming of autoreactive T cells in the periphery (Figure 1C). To confirm the ability of *Camp*^–/–^ mice to mount an effective immune response, we immunized them with OVA and transferred CFSE-labeled OVA-specific CD4^+^ OT-II T cells. Five days after, analyzing OT-II T cell proliferation in the dLNs, we observed similar proliferation regardless of *Camp* deficiency (Supplemental Figure 2). Further supporting that CRAMP was not required for the initiation of the T cell response, 12 days after immunization, the frequency and number of MOG_35-55_-specific CD4^+^ T cells in the SC were similar between WT and *Camp*^–/–^ mice (Figure 1D). However, the expression of IL-17 and IFN-γ in CD4^+^ T cells was reduced in *Camp*^–/–^ mice, supporting a local control of the encephalitogenic T cell response by CRAMP in CNS (Figure 1E). These data underline a role for CRAMP in the promotion of the pathogenic autoreactive CD4^+^ T cell response in the CNS.

To further define the role of CRAMP in EAE, we injected recombinant CRAMP_1-39_ peptide i.p. in WT mice before the symptomatic phase (day 7) or at the peak of the disease (day 15). Surprisingly, the presymptomatic treatment reduced the severity of EAE, and therapeutic treatment at day 15 promoted EAE remission (Figure 1, F and G). Biodistribution experiments showed that Cy5-labeled CRAMP_1-39_ localized to the brain and SC 30 minutes after i.p. injection and remained at these sites for at least 24 hours (Supplemental Figure 3). Similarly, local administration of CRAMP_1-39_ within the intrathecal (i.t.) space of the spine 9 days after EAE induction resulted in a comparable beneficial outcome (Figure 1H). The expression of neurofilament light chain (NF-L) protein in the serum of i.t. CRAMP_1-39_-treated mice on day 15 was decreased compared with untreated mice, indicating reduced neuronal damage (Figure 1I). In addition, i.t. CRAMP_1-39_ treatment decreased the activation of astrocytes and microglia in the SC, as shown by decreased expression of CD44 and CD86, respectively (Figure 1, J and K). The data obtained with CRAMP_1-39_ peptide administration suggest that these AMPs also have protective roles in EAE. We conclude that CRAMP might have antagonistic functions in EAE and proceeded with the investigation of these effects.

### CRAMP is expressed in different cell types within the CNS during EAE.

**Figure 2:**
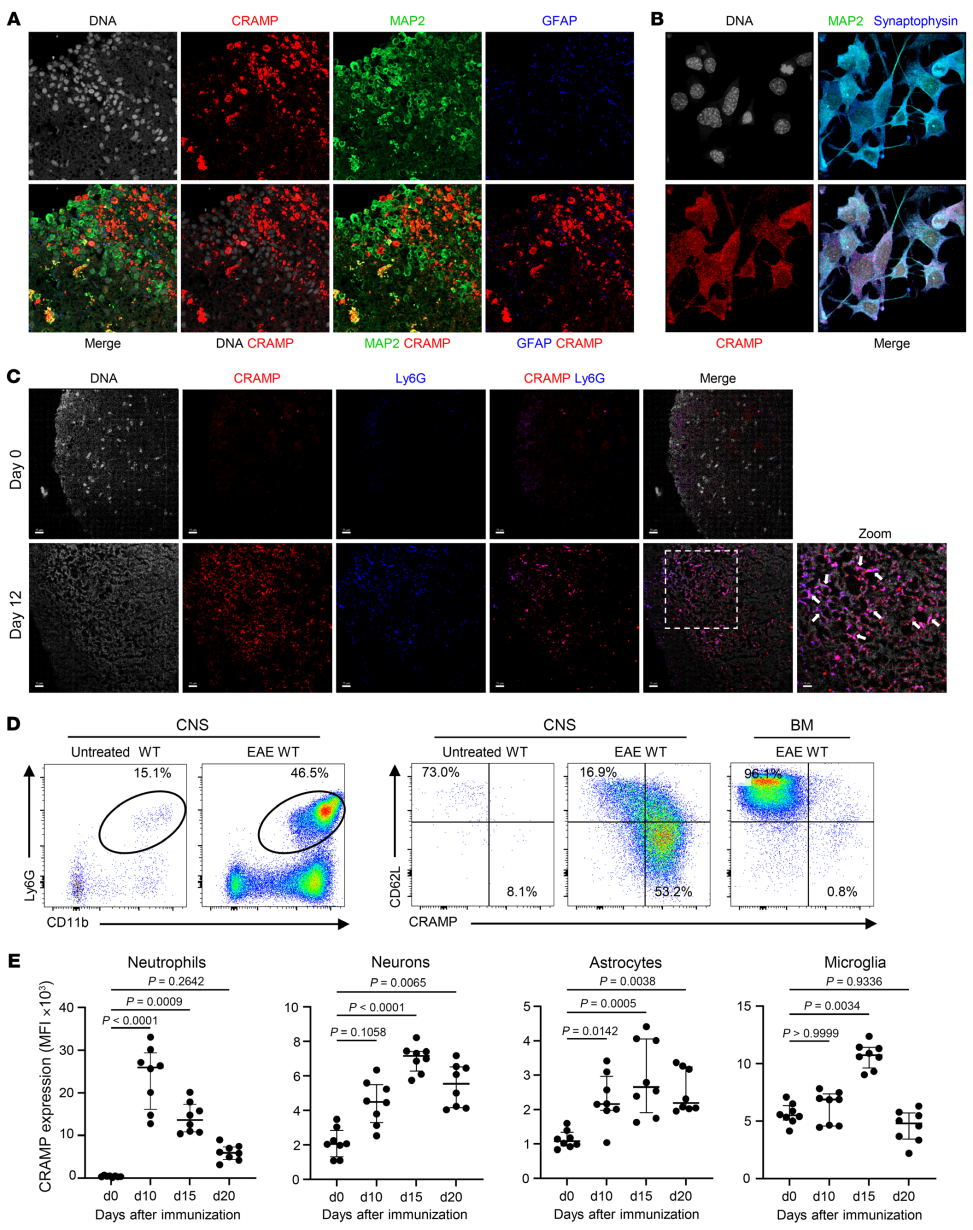
CRAMP is expressed in different cell types within the CNS during EAE. (**A** and **C**) Confocal microscopy images of SC section from WT mice immunized with MOG_35-55_ 12 days earlier. Sections were stained for CRAMP (red), MAP2 (green), GFAP (blue), and DNA (gray) in **A** or for CRAMP (red), Ly6G (blue), and DNA (gray) in **C**. Arrows indicate NETs. Data are representative of 3 independent experiments. Original magnification, ×40. Scale bars: 15 μm. (**B**) Confocal microscopy images of motor neuron-like NSC-34 cells stained for CRAMP (red), MAP2 (green), synaptophysin (blue), and DNA (gray). Data are representative of 3 independent experiments. Original magnification, ×63. (**D** and **E**) Flow cytometry analysis of SC after EAE induction in WT mice. In **D**, 12 days after EAE induction, frequency of neutrophils (CD45^+^CD11b^+^Ly6G^+^Ly6C^lo^) is shown in the left panel and the expression of CRAMP and CD62L by neutrophils is shown in the right panel. BM from tibia rich in immature neutrophils is shown as control. In **E**, the MFI of surface CRAMP expression on neutrophils, neurons (CD45^−^NeuN^+^), astrocytes (CD45^−^GFAP^+^), and microglia (CD45^+^CD11b^+^Tmem119^+^) is shown at different days after EAE induction. Data are the median plus or minus interquartile range of 8 independent mice per group from 3 independent experiments. Comparison between each group was performed using the nonparametric Mann-Whitney *U* test or Kruskal-Wallis test followed up by Dunn’s post test when more than 2 groups were compared.

We envisioned that the opposite effects of CRAMP in EAE might relate to its expression in different cell types. We thus analyzed the expression of CRAMP in the CNS at the peak of EAE using confocal microscopy. CRAMP was expressed in SC by MAP2^+^ neurons and minimally by GFAP^+^ astrocytes, but predominantly by MAP2^–^GFAP^–^ cells (Figure 2A). Neuronal expression of CRAMP was also observed in the NSC-34 motor neuron-like cells (Figure 2B and Supplemental Figure 4A). CRAMP staining colocalized with synaptophysin, a marker of presynaptic vesicles, suggesting that CRAMP can be actively secreted by neurons. It is known that neutrophils infiltrate the CNS early during EAE (27). By confocal microscopy, we confirmed the massive infiltration of the SC by neutrophils after EAE induction. The expression of CRAMP by these neutrophils and the presence of NETs were observed in the SC at day 12 and the dLNs at day 7 (Figure 2C and Supplemental Figure 4, B and C). By flow cytometry, we confirmed the recruitment of CRAMP-expressing activated neutrophils (CD62L^lo^) in the CNS during the initiation of EAE while neutrophils remained nonactivated and did not express CRAMP at their surface in the BM from tibia (Figure 2D). The kinetic of surface CRAMP expression showed high expression in the neutrophils (CD45^+^CD11b^+^Ly6G^+^Ly6C^lo^), peaking at day 10, mild expression in neurons (CD45^–^NeuN^+^) and microglia (CD45^med^CD11b^+^), peaking at day 15, and low expression in astrocytes (CD45^–^GFAP^+^), peaking at day 15 (Figure 2E). These data show that CRAMP was expressed in neutrophils and CNS-resident cells in the CNS during EAE. This was consistent with the hypothesis that the opposite effect of CRAMP on the disease could be explained by different cell sources.

### CRAMP from neutrophils is essential for EAE.

**Figure 3:**
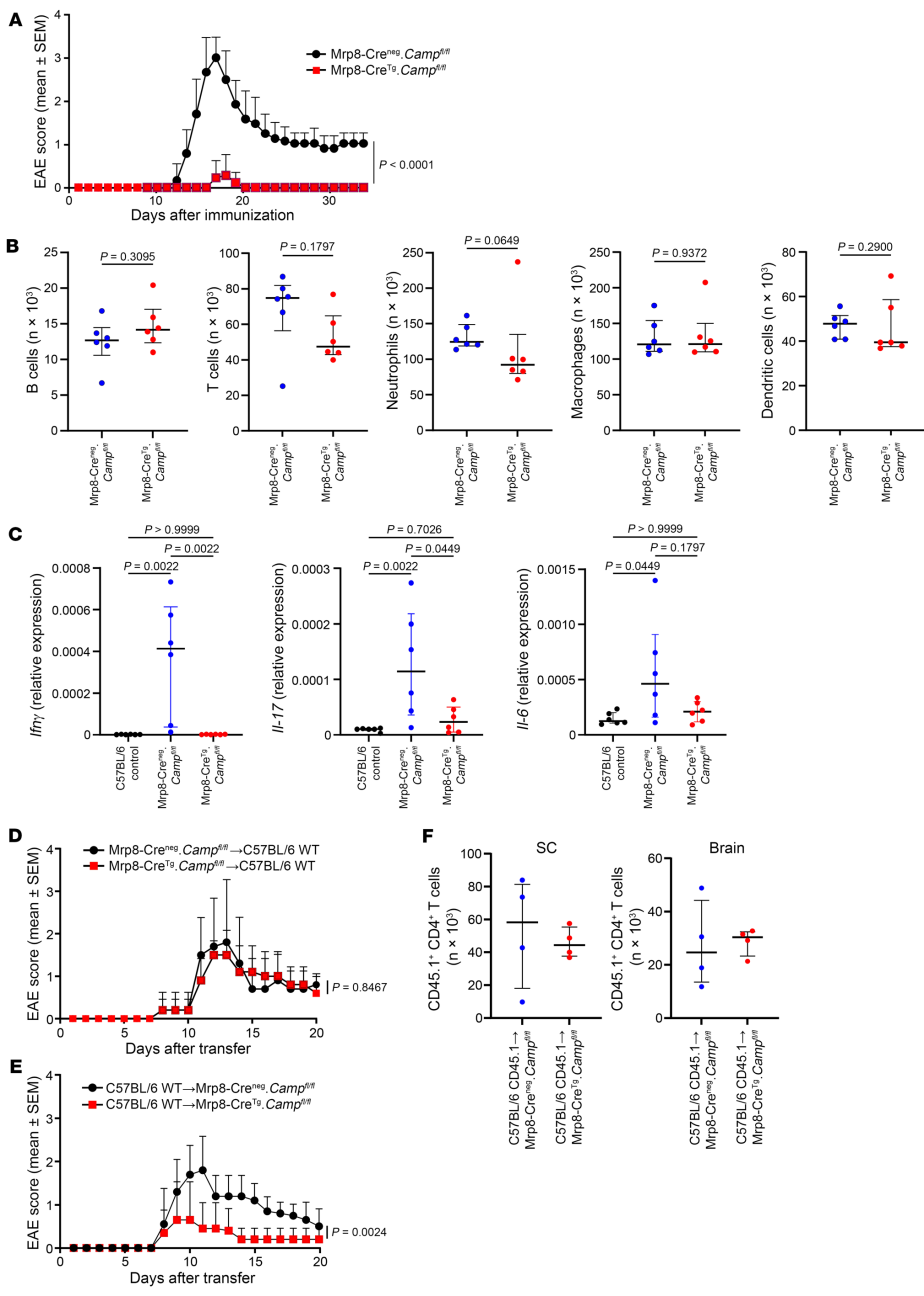
CRAMP from neutrophils is essential for EAE. (**A**) Mrp8-Cre^neg^.*Camp*^fl/fl^ and Mrp8-Cre^Tg^.*Camp*^fl/fl^ mice (*n* = 30 mice per group; 6 independent experiments) were immunized with MOG_35-55_ to induce EAE. Clinical scores are shown (data are represented as mean ± SEM). (**B**) Flow cytometry analysis of SC 15 days after EAE induction. The absolute number of immune cells (CD45^+^), B cells (CD45^+^CD19^+^), T cells (CD45^+^βTCR^+^), neutrophils (CD45^+^CD11b^+^Ly6G^+^Ly6C^lo^), macrophages (CD45^+^CD11b^+^Ly6G^–^F4/80^+^), and dendritic cells (CD45^+^Ly6G^–^F4/80^–^CD11c^+^) is shown. Data are the median plus or minus interquartile range of 6 independent mice per group from 3 independent experiments. (**C**) mRNA expression of cytokines was analyzed by RT-qPCR in SC from Mrp8-Cre^neg^.*Camp*^fl/fl^ and Mrp8-Cre^Tg^.*Camp*^fl/fl^ mice 15 days after EAE induction. Data are the median plus or minus interquartile range of 6 independent mice per group from 3 independent experiments. (**D**−**F**) Splenocytes were recovered 10 days after EAE induction in Mrp8-Cre^neg^.*Camp*^fl/fl^ and Mrp8-Cre^Tg^.*Camp*^fl/fl^ mice (**D**), C57BL/6 WT mice (**E**), or C57BL/6 CD45.1 mice (**F**). Cells were cultured for 3 days with MOG_35-55_ in pro-Th17 conditions and transferred in C57BL/6 WT mice (**D**) or in Mrp8-Cre^neg^.*Camp*^fl/fl^ or Mrp8-Cre^Tg^.*Camp*^fl/fl^ mice (**E** and **F**). Clinical scores are shown (data are represented as mean ± SEM) (*n* = 15 mice per group from 3 independent experiments) in **D** and **E**. Number of CD45.1^+^CD4^+^ T cells in the SC and the brain 7 days after transfer is shown in **F**. Comparison between each group was performed using the nonparametric Mann-Whitney *U* test or Kruskal-Wallis test followed by Dunn’s post test when more than 2 groups were compared. For evaluating differences between EAE scores over time, a 2-way ANOVA with Tukey’s multiple comparisons test was used.

To determine the functional role of CRAMP produced by neutrophils, we generated mice lacking *Camp* specifically in this cell type (Mrp8-Cre^Tg^.*Camp*^fl/fl^ mice) obtained by crossing Mrp8-Cre^Tg^ and *Camp*^fl/fl^ C57BL/6 mice (Supplemental Figure 5, A and B). We observed that Mrp8-Cre^Tg^.*Camp*^fl/fl^ mice were almost resistant to EAE induction, recapitulating the phenotype observed in germline *Camp*^–/–^ mice and demonstrating the mandatory role of CRAMP from neutrophils for EAE development (Figure 3A). Flow cytometry analysis of the immune infiltrate in the SC at the peak of the EAE in Mrp8-Cre^Tg^.*Camp*^fl/fl^ and Mrp8-Cre^neg^.*Camp*^fl/fl^ did not reveal significant differences in terms of cell number or cell types including neutrophils (Figure 3B). However, quantitative reverse transcriptase PCR (RT-qPCR) analysis of the SC showed a reduced expression of the inflammatory cytokines *Ifnγ* and *Il-17*, as well as *Il-6*, in Mrp8-Cre^Tg^.*Camp*^fl/fl^ (Figure 3C). These results suggested a local regulation of disease development that we next confirmed by transfer experiments. Indeed, the transfer of splenocytes from immunized Mrp8-Cre^Tg^.*Camp*^fl/fl^ or Mrp8-Cre^neg^.*Camp*^fl/fl^ mice into C57BL/6 mice induced EAE to the same extent (Figure 3D). However, the transfer of splenocytes from immunized C57BL/6 mice induced EAE in Mrp8-Cre^neg^.*Camp*^fl/fl^ mice but not as effectively in Mrp8-Cre^Tg^.*Camp*^fl/fl^ (Figure 3E), even though the transfer of splenocytes from immunized C57BL/6 CD45.1 mice into Mrp8-Cre^neg^.*Camp*^fl/fl^ or Mrp8-Cre^Tg^.*Camp*^fl/fl^ mice showed no difference in terms of migration of the transferred T cells into the SC and the brain (Figure 3F). These data supported the notion that CRAMP from neutrophils promoted EAE development locally in the CNS rather than in the periphery.

### CRAMP from NETs favors encephalitogenic T cell response.

**Figure 4:**
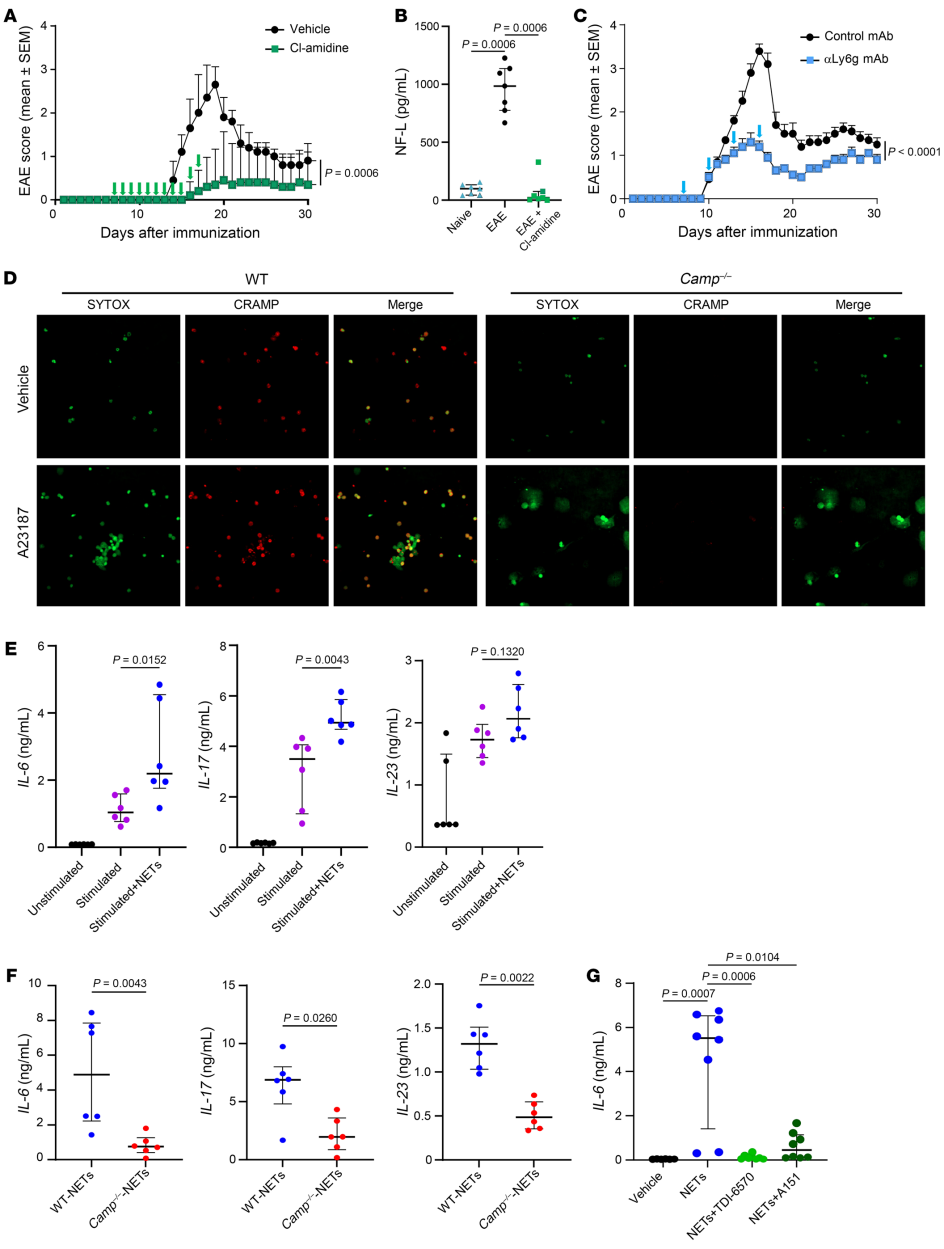
CRAMP from NETs favors encephalitogenic T cell response. (**A** and **C**) WT mice (*n* = 10 mice/group from 2 independent experiments) were immunized with MOG_35-55_ to induce EAE. Mice were then treated s.c. with Cl-amidine daily from day 7 to 17 after EAE induction (**A**) or treated i.p. with neutrophil-depleting αLy6G mAb or isotype control mAb every 3 days from day 7 to 17 after EAE induction (**C**). Clinical scores are shown (mean ± SEM). (**B**) NF-L protein levels were measured in the serum of Cl-amidine-treated mice 15 days after EAE induction. Data are the median plus or minus interquartile range of 7 independent mice per group from 3 independent experiments. (**D**) Neutrophils were isolated from bone marrow of tibia of WT and *Camp*^–/–^ mice and activated with A23187 ionophore to induce NETs. Visualization of NETs was performed by confocal microscopy after DNA (SYTOX green) and CRAMP (red) staining. Data are representative of 3 independent experiments. Original magnification, ×40. (**E** and **F**) NETs were prepared from WT and *Camp*^–/–^ mice as in **E** and added to splenocyte culture isolated from WT mice immunized with MOG_35-55_ 10 days earlier. (**G**) BMDCs from WT mice were stimulated for 18 hours with NETs. In some conditions, the cGAS inhibitor TDI-6570 or a multi-specific TLR9, AIM2, and cGAS antagonist ODN A151 was added to the culture 3 hours before stimulation. Cytokine levels in the supernatant were measured by multiplex ELISA. Data are the median plus or minus interquartile range of 6–8 independent wells from 3 independent experiments. Comparison between each group was performed using the nonparametric Mann-Whitney *U* test or Kruskal-Wallis test followed by Dunn’s post test when more than 2 groups were compared. For evaluating differences between EAE scores over time, a 2-way ANOVA with Tukey’s multiple comparisons test was used.

During EAE, we observed infiltration of CRAMP^+^ NETting neutrophils in the CNS (Figure 2B and Supplemental Figure 4B). Previous studies have shown that NETs contribute via various pathways to the pathogenesis of several autoimmune diseases, including CNS pathologies (28–30). To investigate the role of NETs in EAE development, we administrated the pan-peptidylarginine deiminase (PAD) inhibitor Cl-amidine subcutaneously to WT mice to inhibit NET formation during EAE. We observed that blocking NET formation from day 7 to day 17 after EAE induction significantly reduced disease severity (Figure 4A) and the circulating level of NF-L (Figure 4B). Similarly, neutrophil depletion with anti-Ly6G mAb during the same period also resulted in reduced EAE severity (Figure 4C). To determine the direct effect of NETs on encephalitogenic T cell response, we produced and isolated NETs in vitro from WT and *Camp*^–/–^ neutrophils. We observed that WT and *Camp*^–/–^ neutrophils were able to form NETs similarly (Figure 4D). We added NETs to a culture of splenocytes isolated from EAE-immunized WT mice. WT NETs stimulated the expression of IL-6, IL-23, and IL-17 (Figure 4E), consistent with previous reports demonstrating that NETs promote Th17 response (31). However, CRAMP-deficient NETs failed to promote Th17 response when added to splenocytes from EAE-immunized C57BL/6 mice (Figure 4F). CRAMP is known to promote myeloid immune cell responses to self-nucleic acids released during NETosis by binding and transporting nucleic acids into the cells and enabling their recognition by intracellular sensors such as TLR9, Aim2, or cGAS/STING (15, 32). To confirm this, we generated bone marrow–derived dendritic cells (BMDCs) and stimulated them with NETs in the presence of TDI-6570, a specific cGAS inhibitor, or the ODN A151, a multi-specific TLR9, AIM2, and cGAS antagonist. We observed that NETs stimulated IL-6 from BMDCs and this stimulatory effect was abolished by TDI-6570 and A151 (Figure 4G). Taken together, these data show that CRAMP from CNS-infiltrating neutrophils appeared to be a key molecule promoting EAE development by stimulating the local encephalitogenic Th17 response, possibly via the cGAS/STING pathway.

### CRAMP from neurons dampens EAE.

**Figure 5:**
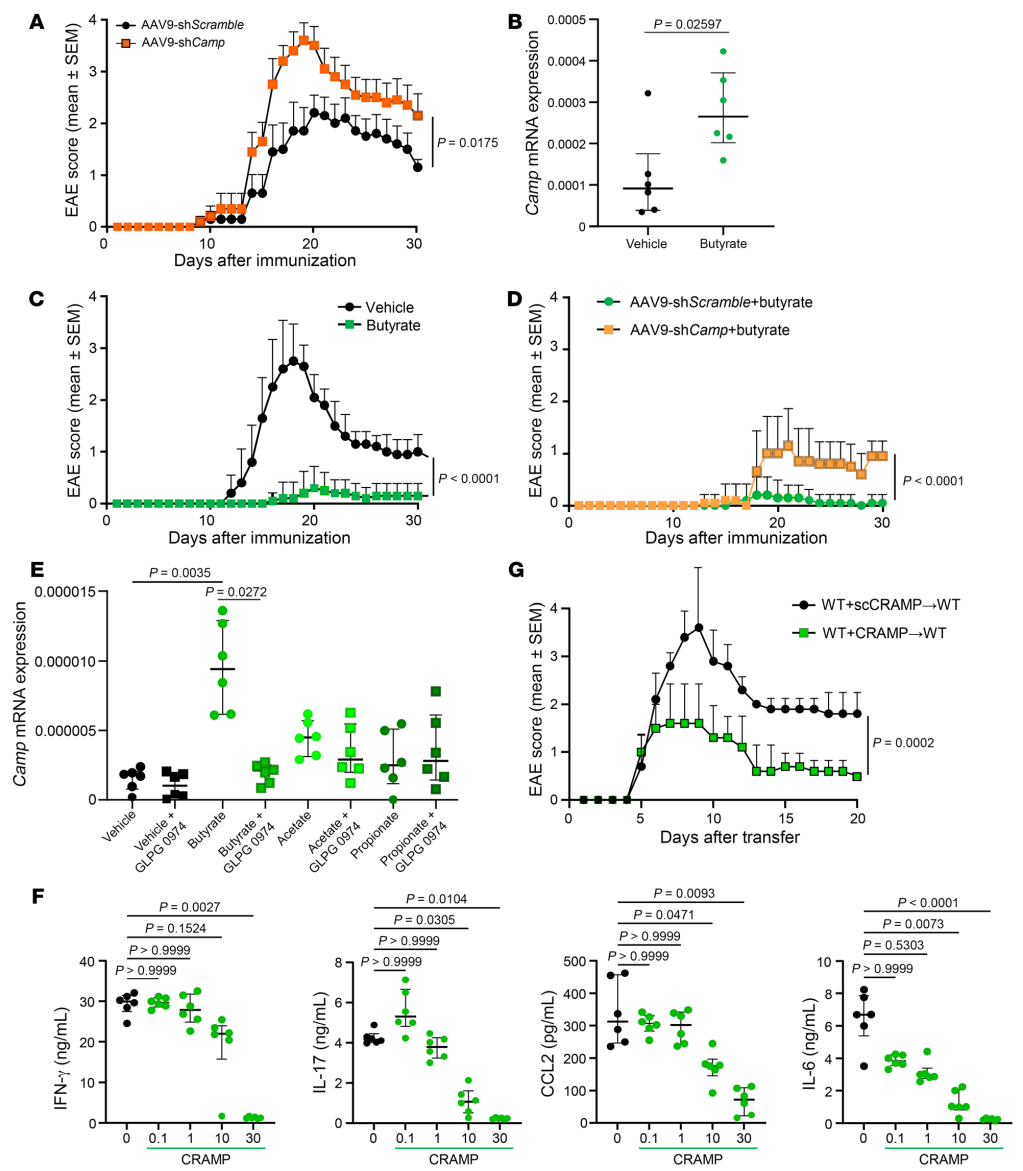
CRAMP from neural cells dampens EAE. (**A**, **C**, and **D**) WT mice were immunized with MOG_35-55_ to induce EAE. C57BL/6 mice were treated with i.t. injection of AAV9-sh*Camp* or AAV9-sh*Scramble* at day –7 (**A**) or with per os (p.o.) butyrate from day 7 to day 30 (**C**) after EAE induction or with both (**D**). Clinical scores are shown (data are represented as mean ± SEM). (*n* = 12 mice per group from 3 independent experiments). (**B**) WT mice were treated with drinking water with sodium butyrate (10 g/L) for 7 days. SC was recovered and *Camp* expression determined by RT-qPCR. Data are the median plus or minus interquartile range of 6 independent mice per group from 3 independent experiments. (**E**) Neuron NSC-34 cells were cultured for 24 hours in the presence of either butyrate, acetate, or propionate analysis of *Camp* expression by RT-qPCR. In some conditions, the FFAR2/3 antagonist GLPG 0974 was added to the cells 2 hours before the SCFAs. Data are the median plus or minus interquartile range of 3 independent experiments. (**F**) Splenocytes were recovered 10 days after EAE induction in WT mice and cultured for 3 days with MOG_35-55_ in pro-Th17 conditions with growing doses of CRAMP_1-39_ (μg/mL). Cytokine levels were measured in the supernatant by multiplex ELISA. Data are the median plus or minus interquartile range of 6 independent mice per group from 3 independent experiments. (**G**) Splenocytes were recovered 10 days after EAE induction in WT mice and were cultured for 3 days with MOG_35-55_ in pro-Th17 conditions with either CRAMP_1-39_ or scCRAMP_1-39_ (10 μg/mL) and transferred in WT mice. Clinical scores are shown (data are represented as mean ± SEM) (*n* = 12 mice/group; 3 independent experiments). Comparisons between groups was performed using the nonparametric Mann-Whitney *U* test or Kruskal-Wallis test followed by Dunn’s post test when more than 2 groups were compared. For evaluating differences between EAE scores over time, a 2-way ANOVA with Tukey’s multiple-comparisons test was used.

As we identified CRAMP expression in neurons, we wanted to determine the functional role of this neural CRAMP on EAE development. To achieve this objective, we performed i.t. injection in WT mice with adeno-associated virus serotype 9 (AAV9) expressing *Camp* shRNA or scramble shRNA sequence under the U6 promoter to knockdown of *Camp* expression by neurons (Supplemental Figure 6). AAV9-sh*Camp* were i.t. injected 7 days before EAE induction in order to selectively reach neurons and not CNS-infiltrating cells. We observed that prophylactic knockdown of *Camp* expression in the SC significantly exacerbated the severity of EAE, supporting a protective role for CRAMP from neurons (Figure 5A). In addition, the oral treatment of WT mice with butyrate, a gut microbiota-derived short chain fatty acid (SCFA) known to induce cathelicidin in several tissues (33), induced CRAMP expression in the CNS at steady state (Figure 5B) and showed protective effect against EAE (Figure 5C). By cell sorting from the CNS, we observed that while butyrate increased *Camp* expression in neurons, microglia, and astrocytes, it decreased *Camp* expression in neutrophils (Supplemental Figure 7A). The protective effect of butyrate involved CRAMP from neurons since butyrate treatment was less effective at reducing EAE severity in mice pretreated with i.t. AAV9-sh*Camp* while it remained protective in mice treated with AAV9-sh*Scramble* (Figure 5D). Other SCFAs, acetate and propionate, also stimulated CRAMP expression in the CNS after oral administration but not significantly (Supplemental Figure 7B). Finally, in vitro experiments using the NSC-34 neuScale rons, showed that in vivo, butyrate, but not acetate or propionate, induced *Camp* expression in neurons. Importantly, using a specific antagonist GLPG 0974, we showed that the stimulatory effect of butyrate on neurons was mediated through the free fatty acid receptor 2/3 (FFAR2/3) receptors (Figure 5E).

To molecularly define the regulatory effect of CRAMP on the encephalitogenic response, we added growing doses of CRAMP_1-39_ on splenocyte culture from EAE-immunized WT mice and we observed a significant reduction in a dose-dependent manner of the expression of IL-17, CCL-2, and IL-6 while IFN-γ expression was poorly impacted (Figure 5F). Notably, these inhibitory effects were observed when CRAMP_1-39_ was applied at 10 μg/mL, which caused little toxicity in the treated cultures, whereas the expression of all cytokines decreased at the dose of 30 μg/mL, because this high concentration of CRAMP_1-39_ killed all cells (Supplemental Figure 8). Accordingly, the transfer of splenocytes from MOG_35-55_-immunized C57BL/6 mice cultured in the presence of CRAMP_1-39_ (10 μg/mL) was less efficient at inducing EAE in C57BL/6 mice compared with control splenocytes not exposed to CRAMP_1-39_ (Figure 5G). Together, these data suggested a model where CRAMP from neurons can control the encephalitogenic response locally.

### CRAMP dampens microglia and astrocyte activation.

**Figure 6:**
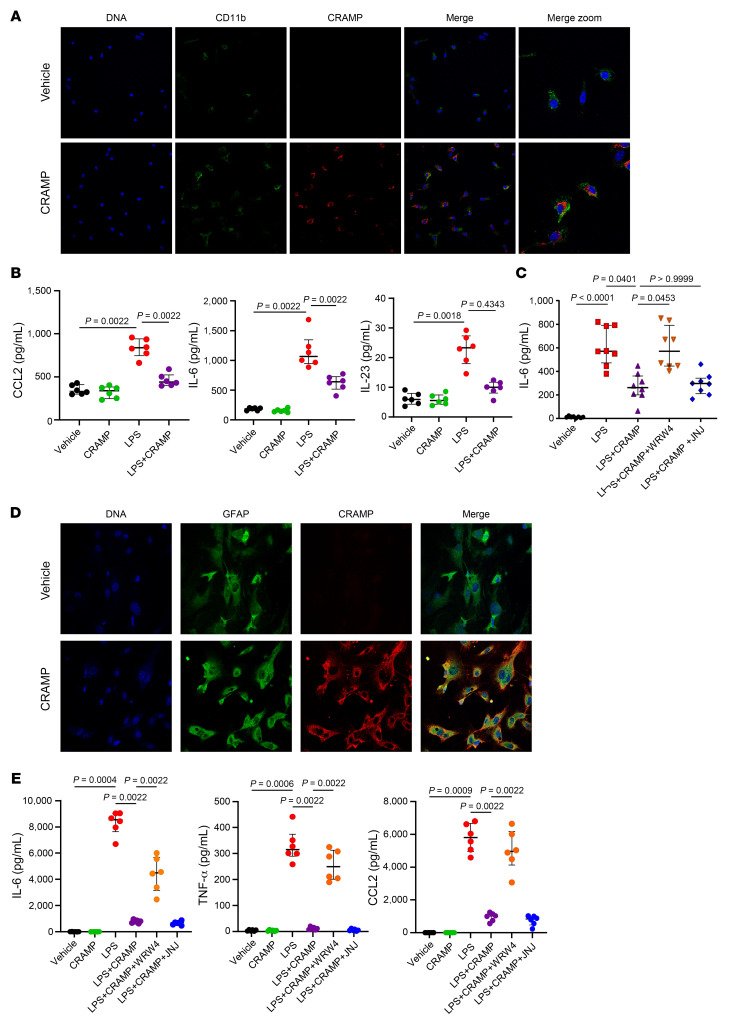
CRAMP dampens microglia and astrocyte activation. (**A**) Microglia culture was prepared from *Camp*^–/–^ mice and 10 μg/mL of CRAMP or vehicle was added for 24 hours before staining for CD11b (green), CRAMP (red), and DNA (blue) and analysis by confocal microscopy. Data are representative of 3 independent experiments. (**B** and **C**) Microglia culture was prepared from WT mice and activated or not with LPS for 24 hours before wash and addition of 10 μg/mL CRAMP_1-39_ or vehicle for an additional 24 hours. In **C**, FPR2 antagonist WRW4 or P2X7R antagonist JNJ-47695567 was added 2 hours before CRAMP_1-39_ addition. Cytokine levels were measured in the supernatant by multiplex ELISA. Data are the median plus or minus interquartile range of 6–8 independent mice per group from 3 independent experiments. (**D**) Astrocyte culture was prepared from *Camp*^–/–^ C57BL/6 mice and 10 μg/mL CRAMP or vehicle was added for 24 hours before staining for GFAP (green), CRAMP (red),and DNA (blue) and analysis by confocal microscopy. Data are representative of 3 independent experiments. Original magnification, ×40. (**E**) Astrocyte cultures were prepared from WT mice and activated or not with LPS for 24 hours before wash and addition of 10 μg/mL CRAMP_1-39_ or vehicle for an additional 24 hours. The FPR2 antagonist WRW4 or P2X7R antagonist JNJ-47695567 was added 2 hours before CRAMP_1-39_ addition. Cytokine levels were measured in the supernatant by multiplex ELISA. Data are the median plus or minus interquartile range of 6 independent mice per group from 3 independent experiments. Comparison between each group was performed using the nonparametric Mann-Whitney *U* test or Kruskal-Wallis test followed by Dunn’s post test when more than 2 groups were compared.

IL-6, IL-23, and CCL2 are critical cytokines and chemokines expressed by microglia, the CNS-resident macrophages (34), and are known to promote the expansion of encephalitogenic Th17 cells during EAE (35). Cathelicidin is known to modulate the phenotype of myeloid immune cells as an example preventing the activation of macrophages by TLR ligands (36). Therefore, we hypothesized that CRAMP prevented microglial activation during EAE, leading to poor Th17 response. To address this question, we isolated primary microglia and cultured them with CRAMP_1-39_. Using microglia from *Camp*^–/–^ mice, we observed that exogenous CRAMP_1-39_ efficiently targeted microglia cells and localized in both their membrane as well as their cytoplasm (Figure 6A). Importantly, CRAMP_1-39_ reduced the production of the pro-Th17 cytokines CCL2, IL-6, and IL-23 by LPS-activated microglia (Figure 6B). By using specific antagonists for receptors known to interact with CRAMP and to be expressed by microglia, we determined that CRAMP_1-39_ mediated its regulatory effect through the formyl peptide receptor 2 (FPR2), rather than the purinergic ionotropic P2X7 receptor (P2X7R) (Figure 6C and Supplemental Figure 9, A and B). In addition, CRAMP_1-39_ also reduced microglial activation induced by a cocktail of IFN-γ and TNF-α, and this regulatory effect was FPR2 dependent (Supplemental Figure 9C). Using primary astrocyte cultures, we demonstrated a similar ability of CRAMP_1-39_ to target this cell type and to decrease inflammatory cytokine expression (IL-6, TNF-α, CCL2) induced by LPS via FPR2 (Figure 6, D and E). These results supported that CRAMP from neurons or exogenous CRAMP_1-39_ had a protective effect against neuroinflammation. It did so by inhibiting the activation of microglia and astrocytes through the proresolving receptor FPR2 and subsequently dampening the encephalitogenic Th17 response.

## Discussion

Our data reveal that CRAMP exerts opposite roles in neuroinflammation depending on its cellular source (Supplemental Figure 10). During EAE initiation, CRAMP is expressed locally by CNS-infiltrating neutrophils. Conditional knockout mice unambiguously show that CRAMP from neutrophils is mandatory for the initiation of EAE. Our in vivo and in vitro experiments demonstrate that NET-associated CRAMP stimulates pro-Th17 response via the cGAS/STING pathway. Recent studies support a role for neutrophils in MS (37). Circulating neutrophils in MS patients show a primed phenotype likely preceding their recruitment in the CNS where they may contribute to local inflammation. In addition, myeloperoxidase (MPO) bound to DNA, which is considered a common marker of NETs, and was found elevated in serum in MS patients (38, 39). In plasma, measures of neutrophil-associated factors NE, CXCL1, and CXCL5 correlate with MS lesion burden and clinical disability (27). Molecular studies identified mRNA from the neutrophil-specific protein ASPRV1 in brain lesions, with higher amounts in severe MS compared with mild or moderate forms, and normal-appearing white matter (40). Neutrophils have been found in the cerebrospinal fluid (CSF) in MS patients during relapse, at an early disease stage, with correlation between the CSF neutrophils and IL-17A levels (41). Pediatric MS patients have neutrophils in the CSF (42), and interestingly, in adults, the neutrophils in the CSF tend to decrease with disease duration (41), both supporting a role for neutrophils locally in early disease. The detrimental role of neutrophils is also documented in 2 MS-related conditions: the neuromyelitis optica spectrum disorder (NMOSD) and the MOG Ab-associated disease (MOGAD). In both diseases, activation of neutrophils occurs in blood circulation, and the production of NETs into inflamed neural tissue is observed in brain from patients (43, 44). In rodent EAE, neutrophil numbers are expanded in the periphery and in the CNS before and during the onset of symptoms (27). Depletion of neutrophils (45–47), inhibition of neutrophil migration by blocking of the neutrophil chemokine receptor CXCR2 (48, 49), and depletion of neutrophil attracting cytokines such as IL-17 (50) and GM-CSF (51) ameliorated the onset and severity of EAE.

The deleterious role of neutrophils in autoimmunity is mostly mediated by their production of NETs. Upon infection, neutrophils release chromatin material and granule content, including AMPs, to form net-like structures known as NETs (52). However, abnormal formation of NETs in sterile context contributes to many autoimmune diseases, including rheumatoid arthritis (RA), systemic lupus erythematosus (SLE), anti-neutrophil cytoplasmic antibody–associated (ANCA-associated) vasculitis, Sjogren’s syndrome (SjS), psoriasis, and type 1 diabetes (T1D) (28). Cathelicidin:nucleic acid complexes in NETs induce inflammatory cytokine production by myeloid immune cells, promoting autoimmune responses (13–15). Human cathelicidin (LL-37) functions as a cargo vehicle to transport extracellular nucleic acids into cells through membrane perturbation, permitting recognition by intracellular recognition systems within the endosome and cytosol such as TLR3, -7, -8, -9 and the cGAS/STING pathway (53, 54). cGAS has been identified as a sensor of NETs in myeloid immune cells, mediating their activation (15). Here, we show that NET-associated CRAMP activates the cGAS/STING pathway in DCs stimulating the secretion of IL-6. It is noteworthy that in vitro, mouse CRAMP and human LL-37 cathelicidin showed ability to bind nucleic acids (55). Mouse CRAMP:nucleic acid complexes can stimulate plasmacytoid DC activation and type I IFN production in the context of skin injury (56), atherosclerosis (57), and autoimmune diabetes (14). Human LL-37:nucleic acid complexes can activate the cGAS/STING pathway in both human THP1 monocyte cells and murine RAW264.7 cells (58). This observation supports that the mechanism described in the mouse EAE model may be relevant to human MS. It has also been demonstrated that cathelicidin from NETs enhances Th17 cell differentiation in dLNs through a direct effect on T cells, contributing to the development of EAE (31, 59). Together, these data support the contribution of CRAMP from NETting neutrophils to EAE, both locally and in the periphery via the promotion of the Th17 response.

Our study also shows that CNS-resident cells, including neurons, express CRAMP, which dampens inflammatory cytokine expression by microglia and astrocytes to control disease severity. The immunoregulatory ability of cathelicidin has been largely documented in various contexts with several studies that have demonstrated that *Camp*^–/–^ mice exhibit a more severe inflammatory phenotype compared with WT mice (60, 61). Cathelicidin and other AMPs modulate the immune response through multiple pathways including interference with TLR signaling, suppression of inflammatory cytokines, or induction of regulatory cytokines (62). In an autoimmune context, cathelicidin from nonimmune cells showed protective effects against RA and T1D through its ability to inhibit locally the expression of inflammatory cytokines by macrophages (63, 64). In the CNS, at steady state, *Camp*^–/–^ mice showed an increased basal level of astrocyte activation, suggesting a role for brain-borne CRAMP in maintaining immune homeostasis (25). In an infectious context, CNS bacterial infection or in vitro stimulation of glial cells by bacterial supernatants increased the expression of CRAMP by astrocytes, microglia, and meningeal cells (21, 24). CNS bacterial infection of *Camp*^–/–^ mice led to a higher degree of glial cell activation accompanied by increased inflammatory cytokine expression in the CNS (25, 26). In the same infectious model, intracerebroventricular infusion of CRAMP_1-39_ led to a decreased expression of inflammatory cytokines, while increasing the expression of the antiinflammatory enzyme HO-1 in the CNS (65). In EAE, a parasitic cathelicidin-like peptide provided disease protection by reducing the expression of TNF-α and IL-6 by macrophages (66). Two recent studies from the same group showed contradictory results regarding the role of cathelicidin in neuroinflammation. They showed that i.t. CRAMP_1-39_ injection (30 μg at day 6 and day 9 after immunization) aggravated EAE and proposed that CRAMP stimulated IFN-γ–primed microglia. It is worth noting that in this study multiple sources of CRAMP in the CNS have also been identified, including neutrophils, astrocytes, neurons, and microglia. However, the relative function of CRAMP from these different cell sources was not deciphered (67). It is important to note that the dose of CRAMP_1-39_ used in this study was at least 3 times higher than the dose we used, potentially causing direct tissue damage (68). In a subsequent study, the same group demonstrated that CRAMP was expressed by astrocytes, neurons, and microglia after in vivo LPS challenge. Intracerebroventricular CRAMP_1-39_ treatment inhibited LPS-mediated neuroinflammation, resulting in decreased expression of inflammatory cytokines. In vitro, consistent with our observations, CRAMP_1-39_ treatment inhibited the expression of inflammatory cytokines by LPS-primed astrocytes and microglia (69).

Our in vitro experiments support that CRAMP_1-39_ inhibits the activation of microglia and astrocytes through the FPR2 receptor. FPR2 is capable of recognizing a wide variety of ligands and has the ability to modulate both pro- and antiinflammatory responses in the CNS, depending on the nature of these ligands (70). FPR2 expression has been described in different brain cell types, where its expression is significantly upregulated by proinflammatory stimuli (71). Activation of FPR2 with various agonists attenuates the effects of neuroinflammatory challenge by reducing the production of proinflammatory cytokines by microglia (72, 73). In line with our data, a recent study showed that treatment of primary murine microglia cells by the FPR2 agonist prevented LPS-induced NF-κB nuclear translocation, which decreased the expression of inflammatory cytokines (74). CRAMP is known to interact with FPR2 in several cell types (75), and our data suggest that CRAMP interaction with FPR2 suppresses inflammatory microglial activity. Taken together, our data and the literature support that the CRAMP:nucleic acid complex released by neutrophils promotes neuroinflammation via the cGAS/STING pathway, whereas the free form of CRAMP secreted by neurons controls neuroinflammation via the proresolving FPR2 expressed by microglia and astrocytes.

Finally, we show that butyrate, a microbiota-derived SCFA, promotes CRAMP expression in neurons via FFAR2/3 receptors, decreased CRAMP expression in neutrophils, and accordingly has a protective role against EAE. Butyrate and other SCFAs are a well-characterized cathelicidin inducer in various rodent and human cell types (76, 77). There is a body of evidence demonstrating that the gut microbiota plays a role in the development of MS and EAE, with altered gut microbiota composition affecting immune function (1, 2, 78). Certain bacterial species contribute to the disease, while others have a dampening effect. Among protective species, butyrate-producing bacteria play a central role in promoting protective Tregs against EAE (79, 80). SCFAs, especially butyrate, promote intestinal Tregs by inhibiting histone deacetylase (HDAC) activity at the *Foxp3* locus (81). Accordingly, butyrate has a protective effect against EAE with fewer Th17 cells in the CNS (82, 83). We propose, as demonstrated by us for T1D (64), that the protective effect of butyrate against EAE is also at least mediated by the stimulation of CRAMP expression by neurons. Butyrate can have EAE-protective effects through various mechanisms. For example, butyrate treatment in aged mice has been shown to promote neurogenesis in the hippocampus (84) and butyrate treatment in a rat model of middle cerebral artery occlusion has been shown to reduce neuronal apoptosis in the CNS via FFAR3 expressed by neurons (85). These results provide valuable insights and suggest potential avenues for future clinical trials targeting MS. Indeed, brain uptake of butyrate and other SCFAs has previously been demonstrated in rats following injection of ^14^C-SCFAs into the carotid artery (86) and a significant concentration of butyrate has been reported in the human brain at steady state (87). Brain concentrations of butyrate can be increased in mice after oral administration of live *Clostridium butyricum* (88), providing a potentially safe therapeutic strategy for MS. Butyrate is a FDA-approved drug and has demonstrated safety and efficacy in humans for treating chronic bacterial infections (89), and several clinical trials are ongoing to test its efficacy against immune-mediated diseases.

## Methods

### Sex as a biological variable.

Female mice were used in this study due to their heightened susceptibility to EAE. However, our findings are expected to be relevant to both sexes, since males develop EAE with a similar physiopathology.

### Mice and treatments.

Female C57BL/6J, C57BL/6J CD45.1, *Camp^−/−^* C57BL/6J, Mrp8-Cre.*Camp*^fl/fl^, and Mrp8-Cre.*Camp*^WT^ C57BL/6J mice between 10 to 14 weeks of ages were used, bred, and housed in specific opportunistic pathogen free conditions in the Necker faculty animal facility. Mrp8-Cre mice were originally purchased from The Jackson Laboratory (Strain 021614) and crossed in our mouse facility. *Camp*^fl/fl^ C57BL/6 mice were generated as follow: *Camp* gene consists of 4 exons on chromosome 9. *Camp*^tm1a(EUCOMM)HMGO^ ES cells (JM8A3.N1; cell clone ID HEPD0722_1_E10; MGl:4950203) targeting the *Camp* locus were purchased from EUCOMM, injected into C57BL/6J embryos, and transferred to recipient female mice. Male chimeric progeny were mated with C57BL/6J female mice to establish germ line transmission. Targeted mice (*Camp*^tm1a(EUCOMM)HMGO^) were then crossed with B6;SJL-Tg(ACTFLPe)9205Dym/J (strain 003800) to generate mice with a “flipped” *Camp* allele lacking the lacZ and neo vector cassettes. *Camp*^fl/fl^ C57BL/6 mice were crossed with neutrophil-specific CRE recombinase line (B6.Cg-Tg(S100A8-cre,-EGFP)1Ilw/J (Mrp8-Cre^Tg^ mice, strain 021614). Breeding of Mrp8-Cre^Tg^.*Camp*^wt/fl^ with *Camp*^fl/fl^ generated mice with excision of *Camp* exons 2–4 in neutrophils (Mrp8-Cre^Tg^.*Camp*^fl/fl^ mice) and littermate controls (Mrp8-Cre^neg^.*Camp*^fl/fl^ mice). Recombinant mouse CRAMP_1–39_ (ISRLAGLLRKGGEKIGEKLKKIGQKIKNFFQKLVPQPEQ) peptide and scrambled CRAMP_1–39_ (scCRAMP_1–39_) (KIGIEQLKGIKGIPEKRPGKRFKLVGEFSNQKALQKLQL) peptide were produced under aseptic conditions and provided after endotoxin removal processing (Innovagen). Peptides were administrated i.p. at a dose of 100 μg diluted in 200 ml of PBS or i.t. at the dose of 10 μg diluted in 10 μl of PBS. Cl-amidine (5 μg/g body weight; Merck) or vehicle (PBS) was injected s.c. from day 7 to 17 after EAE induction. The 1A8 anti-Ly6G mAb was used to deplete neutrophils in EAE mice, whereas the 2A3 mAb served as the isotype control (BioXCell). Abs were administered i.p. 100 μg in 100 μL PBS every 3 days from 7 to 17 days after EAE immunization. Sodium butyrate (Sigma) was administrated in drinking water (10 g/L) for 7 days.

### EAE induction.

EAE was induced by s.c. injections of 200 μg MOG_35–55_ (MEVGWYRSPFSRVVHLYRNGK; SB-peptide) in CFA (AR002, Sigma Aldrich, containing 5 mg/mL heat-inactivated *Mycobacterium tuberculosis* H37Ra [231141, BD Biosciences]). An i.p. injection of 300 ng pertussis toxin (PTX) (Gibco) was also administered on days 0 and 2 of the immunization. The severity of EAE was scored daily using a grading scale of 0–5: 0, unaffected; 0.5, partially limp tail; 1, paralyzed tail; 2, hindlimb paresis and loss in coordinated movement; 2.5, 1 hind limb paralyzed; 3, both hind limbs paralyzed; 3.5, hind limbs paralyzed and weakness in forelimbs; 4, forelimbs paralyzed; 5, death or moribund mice requiring euthanasia. Mice with a grade 2 or above were provided with hydrated food on the floor of the cage.

### i.t. administration of CRAMP_1-39_ and adeno-associated virus Camp shRNA.

AAV9-GFP-U6-m*Camp*-shRNA and AAV9-GFP-U6-scrmb-shRNA (4 × 10^13^ GC/mL) were generated and validated by Vector Biosystems Inc. Mice were injected i.t. with CRAMP_1–39_ peptide (10 μg in 10 μL) or AAVs (10 μL) by a direct lumbar puncture within the i.t. space of the spinal column; this was performed with a 30-gauge needle connected to a 10 μL Hamilton syringe. The puncture of the dura was indicated by a reflexive flick of the tail.

### Preparation of single-cell suspension.

For spleens and lymph nodes, tissues were mashed through a 100 μM strainer and cells were washed with HBSS, 10% FBS, and red blood cells lysed using RBC Lysis Buffer (BioLegend). Immune cells from the SC were isolated using the Multi-Tissue Dissociation Kit 1 (Miltenyi Biotec), and neural cells from the SC or brain were isolated using the Adult Brain Dissociation Kit (Miltenyi Biotec); both were combined with gentleMACS Octo dissociator with heaters according to the manufacturer’s instructions.

### Flow cytometry.

Single-cell suspensions were prepared from various tissues, surface staining was performed after FcγRII/III blocking (anti-CD16/CD32, TruStain FcX, BioLegend) for 5 minutes at 4°C. In all experiments, dead cells were excluded using Fixable Viability Dye (eBioscience). For surface staining, 30 minutes at 4°C, conjugated Abs against the following antigens were used: CD45 (clone 30-F11, BioLegend), CD11c (NF18, BioLegend), CD11b (M1/70, BioLegend), Ly6G (IA8, BioLegend), F4/80 (BM8, BioLegend), CD19 (6D5, BioLegend), TCR-β (H57-597, BioLegend), CD4 (GK1.5, BioLegend), CD44 (IM7, BioLegend), CD86 (GL-1, BioLegend), CD62L (MEL-14, BioLegend, 104428, 1:300), NeuN (3A4C1, Thermo Fisher), GFAP (2.2B10, eBioscience), and Tmem119 (V3RT1Gosz, eBioscience). For intracellular staining, 30 minites at room temperature, conjugated Abs against the following antigens were used: NeuN (3A4C1, Thermo Fisher), and GFAP (2.2B10, eBioscience). Unconjugated Ab for CRAMP (PA-CRPL-100, Innovagen) was used, followed by donkey anti-rabbit IgG (poly4064, BioLegend) staining. MOG_35–55_-specific CD4^+^ T cells were stained using the T-Select I-A^b^ MOG_35–55_ Tetramer-PE (MBL) according to the manufacturer’s instructions. For cytokine expression, cell suspensions were incubated 6 hours at 37°C with cell stimulation cocktail (eBioscience) in the presence of a protein transport inhibitor cocktail (eBioscience), surface stained, fixed, and then intracellularly stained with anti–IL-17A (R&D, 881309) and anti–IFN-γ mAbs (eBioscience, XMG1.2) using the Intracellular Staining Kit (BioLegend).

### RT-qPCR.

Total RNA was isolated using the Nucleospin RNA+ Kit (740984, Macherey-Nagel) from SC. RNA was reverse transcribed to synthesized cDNA using the high-capacity cDNA Reverse Transcription Kit (4368814, Thermo Fisher) and measurements were performed by qPCR using ONEGreen Fast qPCR Premix (OZYA008, Ozyme) on an Azure Cielo 6 Real-time PCR system. Resulting levels of fluorescence were submitted to relative quantification by normalization against a housekeeping gene (GAPDH) and expressed as 2^‒(ΔCT)^ values.

### In vitro NET generation.

Neutrophils were isolated from BM using the Neutrophil Isolation Kit (130-097-658, Miltenyi Biotec) as per the manufacturer’s instructions. Neutrophils were plated at 2 × 10^6^ cells/well in 12-well plates in 500 μL per well in RPMI 2% FBS. 3.8 μM A23187 ionophore (C7522, Sigma) was added to plated neutrophils before incubation for 4 hours at 37°C, 5% CO2 to induce NET formation. Supernatants were carefully discarded, and the remaining NETs were gently washed twice with 1 mL PBS and detached from cells, by digestion with ALU1 (R0137S, NEB) at 4 U/mL in 400 mL per well in RPMI for 20 minutes at 37°C. Digested NETs were collected by mixing vigorously and subsequent centrifugation at 300*g* for 5 minutes at 4°C. Soluble NETs in cell-free supernatant were collected into fresh tubes and stored at −20°C until use. A similar protocol was used to visualize NETs. Neutrophils were plated on round glass coverslips for 2 hours before staining with anti-CRAMP pAb (PA-CRPL, rabbit, Innovagen). After washing, slides were then incubated with donkey anti-rabbit Ab (20966, Biotum). Nucleic acids were stained with SYTOX green (S7020, ThermoFisher).

### Splenocyte culture and transfer.

Single-cell suspension was prepared from spleen 10 days after EAE induction of C57BL/6 mice or in specific experiments of C57BL/6 CD45.1 mice. Cells were cultured for 3 days with 30 μg/mL MOG_35–55_ peptide (SB-peptide) in IMDM 10% FBS, 2 mM l-glutamine, 10 units/mL penicillin, 10 μg/mL streptomycin, 10 mM Hepes, 0.1% sodium pyruvate, 0.1% β-mercaptoethanol (all from Merck) containing 20 ng/mL mouse IL-12, 20 ng/mL mouse IL-23 (all from BioLegend), and 10 μg/mL anti-mouse IFN-γ mAb (BE0055, BioXcell). In some cultures, increasing doses (0.1 to 30 μg/mL) of CRAMP_1–39_ (Innovagen) or NETs (50 μL per 200 μL) were added. For transfer experiments, 300 × 10^6^ cells were cultured in 100 mL, washed, and 50 × 10^6^ cells were injected i.p. in naive mice to induce EAE. The average frequency of CD4^+^ T cells in the culture was 10.9% at day 0 and 15.7% at day 3. For cytokine measurement, 2 × 10^5^ cells were plated per well of a round-bottom 96-well plate. Cytokines were quantitated in the cell culture supernatants with a Legendplex Mouse Inflammation Panel Assay (740446, BioLegend) according to the manufacturer’s instructions.

### BMDC generation and stimulation.

BMDCs were prepared from progenitor cells isolated from the femurs and tibias of 8-week-old female mice. Briefly, BM cells were plated on 6-well low-cluster plates in RPMI 1640 medium containing 10% FCS and 1% penicillin/streptomycin and supplemented with 10 ng/mL murine GM-CSF (R&D Systems) for 8 days. In some conditions, NETs (50 μL per 500 μL), cGAS inhibitor TDI-6570 (10 μM, Invivogen), or multi-specific TLR9, AIM2, and cGAS antagonist ODN A151 (10 μM, Invivogen) were added 18 hours before analysis. Cytokines were quantitated in the cell culture supernatants with a Legendplex Mouse Inflammation Panel Assay (BioLegend) according to the manufacturer’s instructions.

### OVA immunization and proliferation of OT-II T cells.

CD4^+^ OT-II T cells were isolated from C57BL/6-Tg(TcraTcrb)425Cbn/Crl mice using the naive CD4 T Cell Isolation Kit (130-104-454, Miltenyi Biotech) and labeled with CellTrace CFSE (C34554, Thermo Fisher Scientific) according to the manufacturer’s instructions. OT-II cells were transferred i.v. 1 day prior to immunization with 100 μg OVA emulsified in CFA in rear footpads. Seven days later, single-cell suspensions generated from the dLNs and OT-II cell division were analyzed by flow cytometry.

### Immunofluorescence staining.

The SC and cervical lymph nodes were isolated and frozen in OCT (4583, Sakura Finetek) in liquid nitrogen to be cryo-sectioned (5 μM). Frozen sections were air dried for 10 minutes before fixation in 4% PFA for an additional 10 minutes. Slides were permeabilized with PBS 0.1% Triton X-100 (Sigma) and blocked with commercial blocking buffer (ab64226, Abcam) for 1 hour at room temperature (RT), then primary Abs were incubated in 10% donkey serum overnight at 4°C. Primary Abs used were as follows: CRAMP (PA-CRPL, rabbit, Innovagen), anti-Ly6G (NIMP-R14, rat, Abcam), MAP2 (AP-20, mouse, Abcam), and GFAP (ab4674, chicken, Abcam). After washing, slides were then incubated with a combination of the following secondary Abs coupled with CF dyes: donkey anti-rat (catalog 20843), donkey anti-rabbit (catalog 20966), donkey anti-chicken (catalog 20166), and donkey anti-mouse (catalog 20014) (all from Biotum). Nuclei were stained with DAPI (D1306, Molecular probes). Image acquisition was performed on SFR Necker Imaging Facility using a Zeiss Spinning disk microscope.

### Primary glial cell culture.

For primary microglial cell cultures, the brains of 3-day-old mice were homogenized and mechanically disrupted with a nylon mesh and filtered through a 70 μM cell strainer. The obtained mixed glial cells were seeded in culture flasks and grown in an incubator at 37°C with 5% CO_2_ in DMEM, 10% FBS, 200 mM l-glutamine, 100 U/mL penicillin, 0.1 mg/mL streptomycin, and OPI supplement (oxalacetic acid, pyruvate, insulin; O5003, Sigma). Culture media was changed initially at day 5 and then every 3 days. After 14 days of culture, primary microglia were obtained from mixed glial cells by shaking overnight at 200 rpm at 37°C and maintained in complete medium. Cells were activated with LPS for 24 hours before the addition of 10 μg/mL CRAMP_1–39_ or vehicle for an additional 24 hours. Astrocytes were isolated from adult brain using the Adult Brain Dissociation Kit (130-107-667, Miltenyi Biotec) according to the manufacturer’s instructions. Isolated astrocytes, 10^5^ cells, were maintained in culture in 24-well plate in MACS Neuro Medium containing 2% MACS Neuro Brew-21, 1% penicillin/streptomycin, and 0.5 mM l-glutamine. Cells were activated with LPS for 24 hours before addition of 10 μg/mL CRAMP_1–39_ or vehicle for an additional 24 hours. In some conditions, FPR2 antagonist WRW4 or P2X7R antagonist JNJ-47695567 (both from Tocris Bioscience) was added 2 hours before CRAMP addition. Cytokines were quantitated in the cell culture supernatants with a Legendplex Mouse Macrophage Panel Assay (740846, Biolegend) according to the manufacturer’s instructions.

### In vitro culture of NSC-34 neuron cell line.

For neuron differentiation, NSC-34 cells (CLU140-A, Tebubio) were seeded at a concentration of 5 × 10^4^ cells per cm² in collagen-coated plates. The proliferation medium (DMEM, 10% FBS, 100 U/mL penicillin, 0.1 mg/mL streptomycin) was replaced 24 hours after seeding by differentiation medium (DMEM/F-12 [ham], 1% FBS, 1% modified Eagle’s medium nonessential amino acids, and 1 μM retinoic acid [RA, #554720, Sigma]). Cells were allowed to differentiate for 4 days. In some experiments, CRAMP expression was analyzed by confocal microscopy as described in the *Immunofluorescence staining* section. Primary Abs used were as follows: CRAMP (PA-CRPL, rabbit, Innovagen), MAP2 (ab5392, chicken, Abcam), and synaptophysin (SP11, mouse, Thermo Fisher). After washing, slides were then incubated with a combination of the following secondary Abs coupled with CF dyes: donkey anti-rabbit (catalog 20966), donkey anti-chicken (catalog 20166), and donkey anti-mouse (catalog 20046) (all from Biotum). Nuclei were stained with DAPI (D1306, Molecular Probes). In some experiments, the differentiation medium was replaced by RA-free medium and cells were stimulated for 24 hours by 10 μg/mL of each SCFA: sodium butyrate, sodium acetate, or sodium propionate. The GRP43 antagonist GLPG 0974 (1 μM, Tocris) was added to the culture 2 hours before the addition of the SCFAs. Then *Camp* mRNA expression was analyzed by RT-qPCR.

### Statistics.

Comparison between each group was performed using the nonparametric Mann-Whitney *U* test or Kruskal-Wallis test followed by Dunn’s post test when more than 2 groups were compared. For evaluating differences between EAE scores over time, a 2-way ANOVA with Tukey’s multiple-comparisons test was used. *P* values < 0.05 were considered statistically significant. All data were analyzed using GraphPad Prism, version 9, software.

### Study approval.

All animal experimental protocols were approved by the French Ethics Committee for Animal Experimentation (APAFIS 19833-2018031214353174).

### Data availability.

Values for all data points in graphs are reported in the Supporting Data Values file.

## Author contributions

SCV and EE performed and analyzed experiments, interpreted data, and wrote the paper with general assistance of JD. FR, KM, CBT, and AP performed specific in vitro experiments. MT performed biodistribution experiments. DRG provided expertise in AAV-shRNA design and i.t. injection. SF and FO provided intellectual input and expertise to analyze experiments and wrote the paper. RL and JD designed the project, interpreted data, and wrote the paper. Authorship order among the 2 co–first authors was determined by who initiated the work.

## Supplementary Material

Supplemental data

Supporting data values

## Version 1 12/10/2024

In-Press Preview

## Version 2 02/03/2025

Electronic publication

## References

[1] Berer K (2011). Commensal microbiota and myelin autoantigen cooperate to trigger autoimmune demyelination. Nature.

[2] iMSMS Consortium (2022). Gut microbiome of multiple sclerosis patients and paired household healthy controls reveal associations with disease risk and course. Cell.

[3] Berger JR, Markowitz C (2018). Deciding on the best multiple sclerosis therapy: tough choices. JAMA Neurol.

[4] Fan Y, Zhang J (2019). Dietary modulation of intestinal microbiota: future opportunities in experimental autoimmune encephalomyelitis and multiple sclerosis. Front Microbiol.

[5] Codarri L (2013). Communication between pathogenic T cells and myeloid cells in neuroinflammatory disease. Trends Immunol.

[6] Ransohoff RM (2012). Animal models of multiple sclerosis: the good, the bad and the bottom line. Nat Neurosci.

[7] Mrdjen D (2018). High-dimensional single-cell mapping of central nervous system immune cells reveals distinct myeloid subsets in health, aging, and disease. Immunity.

[8] Hultmark D (1980). Insect immunity. Purification and properties of three inducible bactericidal proteins from hemolymph of immunized pupae of Hyalophora cecropia. Eur J Biochem.

[9] Gallo RL, Hooper LV (2012). Epithelial antimicrobial defence of the skin and intestine. Nat Rev Immunol.

[10] Liang WJ (2022). Intestinal cathelicidin antimicrobial peptide shapes a protective neonatal gut microbiota against pancreatic autoimmunity. Gastroenterology.

[11] Hancock RE (2016). The immunology of host defence peptides: beyond antimicrobial activity. Nat Rev Immunol.

[12] Pinegin B (2015). Neutrophil extracellular traps and their role in the development of chronic inflammation and autoimmunity. Autoimmun Rev.

[13] Lande R (2011). Neutrophils activate plasmacytoid dendritic cells by releasing self-DNA-peptide complexes in systemic lupus erythematosus. Sci Transl Med.

[14] Diana J (2013). Crosstalk between neutrophils, B-1a cells and plasmacytoid dendritic cells initiates autoimmune diabetes. Nat Med.

[15] Apel F (2021). The cytosolic DNA sensor cGAS recognizes neutrophil extracellular traps. Sci Signal.

[16] Liang W, Diana J (2020). The dual role of antimicrobial peptides in autoimmunity. Front Immunol.

[17] Su Y (2010). Antimicrobial peptides in the brain. Arch Immunol Ther Exp (Warsz).

[18] Stuart BAR (2022). Regulatory roles of antimicrobial peptides in the nervous system: implications for neuronal aging. Front Cell Neurosci.

[19] Bergman P (2005). The antimicrobial peptide rCRAMP is present in the central nervous system of the rat. J Neurochem.

[20] Bergman P (2006). Induction of the antimicrobial peptide CRAMP in the blood-brain barrier and meninges after meningococcal infection. Infect Immun.

[21] Brandenburg LO (2008). Role of glial cells in the functional expression of LL-37/rat cathelin-related antimicrobial peptide in meningitis. J Neuropathol Exp Neurol.

[22] Williams WM (2012). Do beta-defensins and other antimicrobial peptides play a role in neuroimmune function and neurodegeneration?. ScientificWorldJournal.

[23] Appelgren D (2019). Neutrophil extracellular traps (NETs) in the cerebrospinal fluid samples from children and adults with central nervous system infections. Cells.

[24] Brandenburg LO (2009). Expression and regulation of antimicrobial peptide rCRAMP after bacterial infection in primary rat meningeal cells. J Neuroimmunol.

[25] Merres J (2014). Role of the cathelicidin-related antimicrobial peptide in inflammation and mortality in a mouse model of bacterial meningitis. J Innate Immun.

[26] Kress E (2017). CRAMP deficiency leads to a pro-inflammatory phenotype and impaired phagocytosis after exposure to bacterial meningitis pathogens. Cell Commun Signal.

[27] Rumble JM (2015). Neutrophil-related factors as biomarkers in EAE and MS. J Exp Med.

[28] Wigerblad G, Kaplan MJ (2023). Neutrophil extracellular traps in systemic autoimmune and autoinflammatory diseases. Nat Rev Immunol.

[29] Shafqat A (2023). Neutrophil extracellular traps in central nervous system pathologies: a mini review. Front Med (Lausanne).

[30] Geeraerts T (2023). Immunohistologic features of cerebral venous thrombosis due to vaccine-induced immune thrombotic thrombocytopenia. Neurol Neuroimmunol Neuroinflamm.

[31] Minns D (2021). The neutrophil antimicrobial peptide cathelicidin promotes Th17 differentiation. Nat Commun.

[32] Alford MA (2020). Cathelicidin host defense peptides and inflammatory signaling: striking a balance. Front Microbiol.

[33] Kida Y (2006). Sodium butyrate up-regulates cathelicidin gene expression via activator protein-1 and histone acetylation at the promoter region in a human lung epithelial cell line, EBC-1. Mol Immunol.

[34] Jahan-Abad AJ (2020). Serum pro-inflammatory and anti-inflammatory cytokines and the pathogenesis of experimental autoimmune encephalomyelitis. Neuropathology.

[35] Ji Z (2019). Obesity promotes EAE through IL-6 and CCL-2-mediated T cells infiltration. Front Immunol.

[36] Scheenstra MR (2020). Cathelicidins modulate TLR-activation and inflammation. Front Immunol.

[37] Woodberry T (2018). The emerging role of neutrophil granulocytes in multiple sclerosis. J Clin Med.

[38] Naegele M (2012). Neutrophils in multiple sclerosis are characterized by a primed phenotype. J Neuroimmunol.

[39] Minohara M (2006). Upregulation of myeloperoxidase in patients with opticospinal multiple sclerosis: positive correlation with disease severity. J Neuroimmunol.

[40] Whittaker Hawkins RF (2017). ICAM1+ neutrophils promote chronic inflammation via ASPRV1 in B cell-dependent autoimmune encephalomyelitis. JCI Insight.

[41] Kostic M (2014). IL-17 and glutamate excitotoxicity in the pathogenesis of multiple sclerosis. Scand J Immunol.

[42] Chabas D (2010). Younger children with MS have a distinct CSF inflammatory profile at disease onset. Neurology.

[43] Murata H (2022). Cell-free DNA derived from neutrophils triggers type 1 interferon signature in neuromyelitis optica spectrum disorder. Neurol Neuroimmunol Neuroinflamm.

[44] Hoftberger R (2020). The pathology of central nervous system inflammatory demyelinating disease accompanying myelin oligodendrocyte glycoprotein autoantibody. Acta Neuropathol.

[45] Aube B (2014). Neutrophils mediate blood-spinal cord barrier disruption in demyelinating neuroinflammatory diseases. J Immunol.

[46] McColl SR (1998). Treatment with anti-granulocyte antibodies inhibits the effector phase of experimental autoimmune encephalomyelitis. J Immunol.

[47] Shen P (2023). Toll-like receptors control the accumulation of neutrophils in lymph nodes that expand CD4^+^ T cells during experimental autoimmune encephalomyelitis. Eur J Immunol.

[48] Carlson T (2008). The Th17-ELR+ CXC chemokine pathway is essential for the development of central nervous system autoimmune disease. J Exp Med.

[49] Liu Y (2015). Preferential recruitment of neutrophils into the cerebellum and brainstem contributes to the atypical experimental autoimmune encephalomyelitis phenotype. J Immunol.

[50] Komiyama Y (2006). IL-17 plays an important role in the development of experimental autoimmune encephalomyelitis. J Immunol.

[51] McQualter JL (2001). Granulocyte macrophage colony-stimulating factor: a new putative therapeutic target in multiple sclerosis. J Exp Med.

[52] Brinkmann V (2004). Neutrophil extracellular traps kill bacteria. Science.

[53] Zielke C (2024). Between good and evil: Complexation of the human cathelicidin LL-37 with nucleic acids. Biophys J.

[54] Takahashi T (2018). Cathelicidin promotes inflammation by enabling binding of self-RNA to cell surface scavenger receptors. Sci Rep.

[55] Wong A (2018). A novel biological role for peptidyl-arginine deiminases: citrullination of cathelicidin LL-37 controls the immunostimulatory potential of cell-free DNA. J Immunol.

[56] Gregorio J (2010). Plasmacytoid dendritic cells sense skin injury and promote wound healing through type I interferons. J Exp Med.

[57] Doring Y (2012). Auto-antigenic protein-DNA complexes stimulate plasmacytoid dendritic cells to promote atherosclerosis. Circulation.

[58] Wei X (2022). LL-37 transports immunoreactive cGAMP to activate STING signaling and enhance interferon-mediated host antiviral immunity. Cell Rep.

[59] Smith KJ (2022). The antimicrobial peptide cathelicidin drives development of experimental autoimmune encephalomyelitis in mice by affecting Th17 differentiation. PLoS Biol.

[60] Deng YY (2016). Cathelicidin-related antimicrobial peptide modulates the severity of acute pancreatitis in mice. Mol Med Rep.

[61] Severino P (2017). Cathelicidin-deficient mice exhibit increased survival and upregulation of key inflammatory response genes following cecal ligation and puncture. J Mol Med (Berl).

[62] Mookherjee N (2020). Antimicrobial host defence peptides: functions and clinical potential. Nat Rev Drug Discov.

[63] Chow LN (2014). Human cathelicidin LL-37-derived peptide IG-19 confers protection in a murine model of collagen-induced arthritis. Mol Immunol.

[64] Sun J (2015). Pancreatic β-cells limit autoimmune diabetes via an immunoregulatory antimicrobial peptide expressed under the influence of the gut microbiota. Immunity.

[65] Dorr A (2015). Intrathecal application of the antimicrobial peptide CRAMP reduced mortality and neuroinflammation in an experimental model of pneumococcal meningitis. J Infect.

[66] Lund ME (2016). A parasite-derived 68-mer peptide ameliorates autoimmune disease in murine models of type 1 diabetes and multiple sclerosis. Sci Rep.

[67] Bhusal A (2022). Cathelicidin-related antimicrobial peptide promotes neuroinflammation through astrocyte-microglia communication in experimental autoimmune encephalomyelitis. Glia.

[68] Pinheiro da Silva F (2009). Differing effects of exogenous or endogenous cathelicidin on macrophage toll-like receptor signaling. Immunol Cell Biol.

[69] Bhusal A (2022). Cathelicidin-related antimicrobial peptide negatively regulates bacterial endotoxin-induced glial activation. Cells.

[70] Tylek K (2021). Formyl peptide receptor 2, as an important target for ligands triggering the inflammatory response regulation: a link to brain pathology. Pharmacol Rep.

[71] Pourbadie HG (2018). Early minor stimulation of microglial TLR2 and TLR4 receptors attenuates Alzheimer’s disease-related cognitive deficit in rats: behavioral, molecular, and electrophysiological evidence. Neurobiol Aging.

[72] Slowik A (2012). Involvement of formyl peptide receptors in receptor for advanced glycation end products (RAGE)--and amyloid beta 1-42-induced signal transduction in glial cells. Mol Neurodegener.

[73] Tylek K (2021). Time-dependent protective and pro-resolving effects of FPR2 agonists on lipopolysaccharide-exposed microglia cells involve inhibition of NF-κB and MAPKs pathways. Cells.

[74] Wickstead ES (2023). Stimulation of the pro-resolving receptor Fpr2 reverses inflammatory microglial activity by suppressing NFκB activity. Int J Mol Sci.

[75] Jeong YS, Bae YS (2020). Formyl peptide receptors in the mucosal immune system. Exp Mol Med.

[76] Schauber J (2003). Expression of the cathelicidin LL-37 is modulated by short chain fatty acids in colonocytes: relevance of signalling pathways. Gut.

[77] Steinmann J (2009). Phenylbutyrate induces antimicrobial peptide expression. Antimicrob Agents Chemother.

[78] Correale J (2022). The role of the gut microbiota in multiple sclerosis. Nat Rev Neurol.

[79] Haghikia A (2015). Dietary fatty acids directly impact central nervous system autoimmunity via the small intestine. Immunity.

[80] Bianchimano P (2022). Mining the microbiota to identify gut commensals modulating neuroinflammation in a mouse model of multiple sclerosis. Microbiome.

[81] Arpaia N (2013). Metabolites produced by commensal bacteria promote peripheral regulatory T-cell generation. Nature.

[82] Mizuno M (2017). The dual role of short fatty acid chains in the pathogenesis of autoimmune disease models. PLoS One.

[83] Wang C (2022). Methyl butyrate alleviates experimental autoimmune encephalomyelitis and regulates the balance of effector T Cells and regulatory T cells. Inflammation.

[84] Kundu P (2019). Neurogenesis and prolongevity signaling in young germ-free mice transplanted with the gut microbiota of old mice. Sci Transl Med.

[85] Zhou Z (2021). Sodium butyrate attenuated neuronal apoptosis via GPR41/Gβγ/PI3K/Akt pathway after MCAO in rats. J Cereb Blood Flow Metab.

[86] Oldendorf WH (1973). Carrier-mediated blood-brain barrier transport of short-chain monocarboxylic organic acids. Am J Physiol.

[87] Bachmann C (1979). Short chain fatty acids in plasma and brain: quantitative determination by gas chromatography. Clin Chim Acta.

[88] Liu J (2015). Neuroprotective effects of clostridium butyricum against vascular dementia in mice via metabolic butyrate. BioMed Res Int.

[89] Chen J, Vitetta L (2020). The role of butyrate in attenuating pathobiont-induced hyperinflammation. Immune Netw.

