# Supplementary material for: Cathelicidin antimicrobial peptide expression in neutrophils and neurons antagonistically modulates neuroinflammation
Source: J Clin Invest. 2024 Dec 10;135(3):e184502. doi: 10.1172/JCI184502 (PMC11785927; doi:10.1172/JCI184502)
Supplement: Supplemental data [file jci-135-184502-s050.pdf]

Supplemental Figure 1

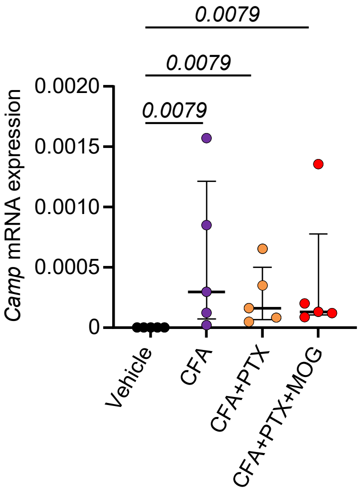

Figure S1 Induction of *Camp* expression in the CNS of mice after administration of the different components of the EAE-immunization cocktail. mRNA expression of *Camp* was analyzed by RT-qPCR in spinal cord from C57BL/6 WT mice 12 days after treatment with complete Freund's adjuvant (CFA, containing 5 mg.mL-1 heat-inactivated Mycobacterium tuberculosis H37Ra, 200 µL in emulsion with PBS, subcutaneously), CFA and pertussis toxin (PTX, 300 ng in PBS, intraperitoneally on days 0 and 2 of the immunization), or the combination of CFA, PTX and MOG_{35-55} peptide (200 µg in emulsion with the CFA) as described in the Methods. Data are the median +/− interquartile range of 5 independent mice per group from 2 independent experiments.

Supplemental Figure 2

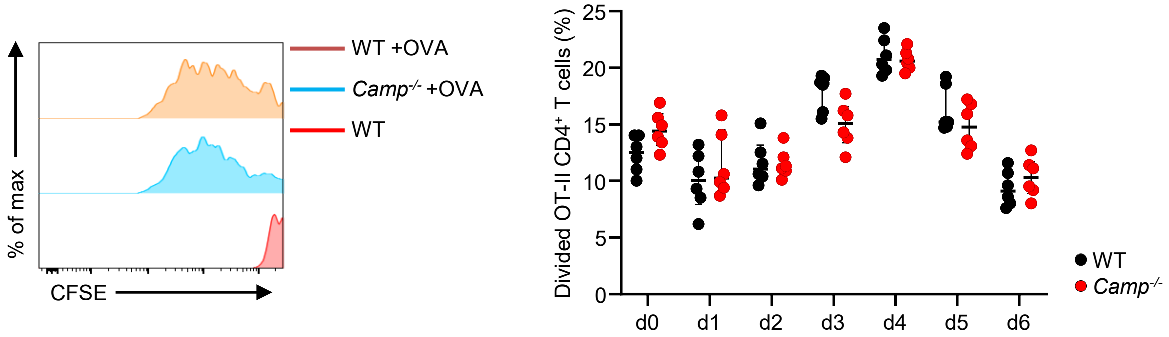

Figure S2 Priming of OVA-specific CD4 T cells in the periphery was similar regardless of CRAMP deficiency. CFSE-labeled OT-II OVA-specific $CD4^{+}$ T cells were isolated and transferred into WT and *Camp*$^{-/-}$ mice. Mice were immunized mice with ovalbumin (OVA) 24h later and OT-II cells proliferation was analyzed by flow cytometry measuring CFSE dilution 7 days post-immunization. The frequency of cells in each consecutive cell division is shown. Data are the median +/− interquartile range of 6 independent mice per group.

Supplemental Figure 3

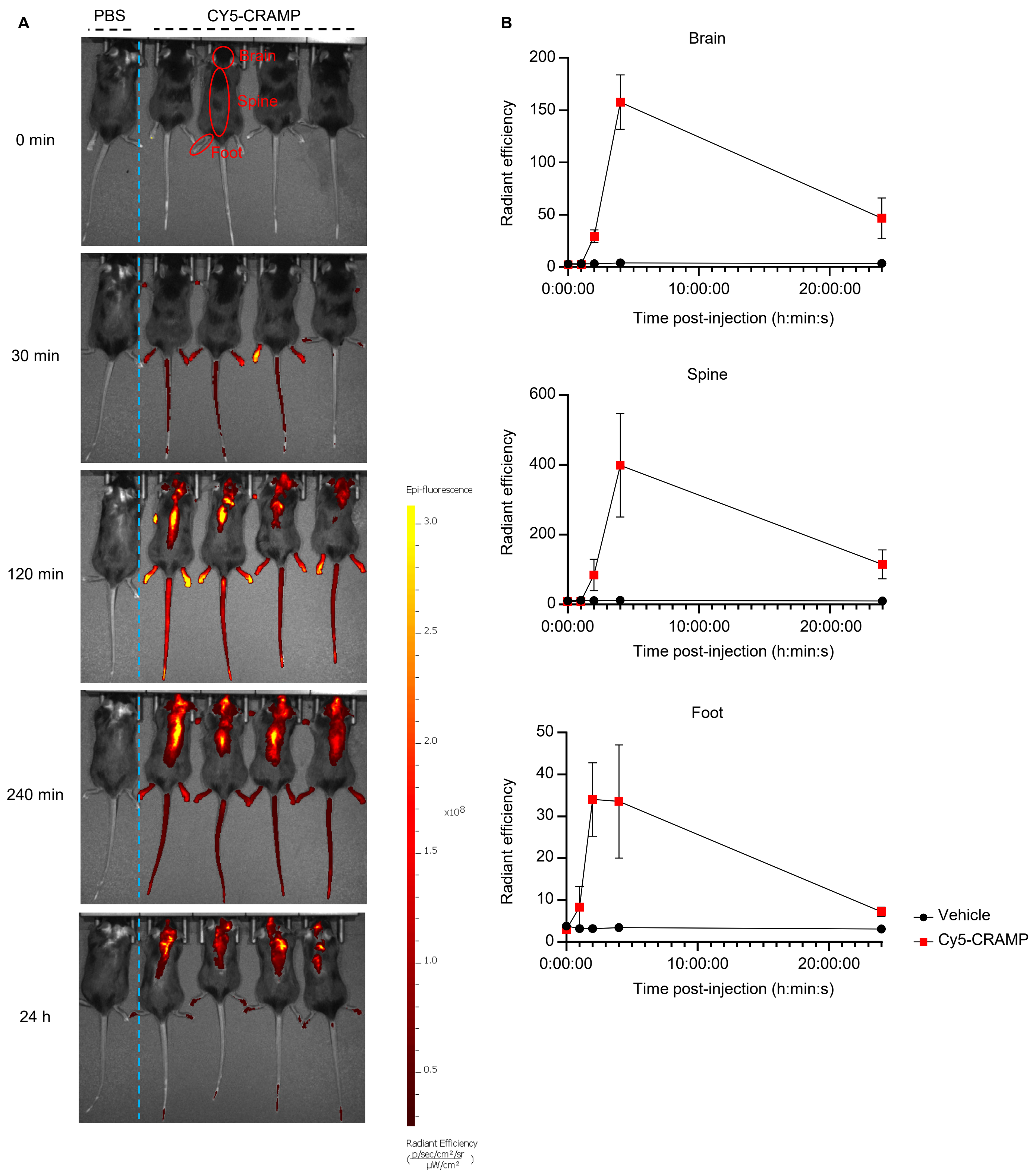

Figure S3 Biodistribution of CRAMP after intraperitoneal injection. Cyanin5-labelled CRAMP$_{1\text{-}39}$ peptide (20 µg, SB-peptide) or PBS was injected intraperitoneally in C57BL/6 mice previously treated with pertussis toxin (300 ng, i.p.) at day -2 and -7. Anesthetized mice were imaged up to 24 h to follow fluorescence accumulation in tissues using an IVIS Spectrum CT system (Perkin Elmer). (**A**) Representative images show fluorescence (radiant efficiency) in the defined regions of interest (ROI, red circles: brain, spinal cord and foot). (**B**) Quantification of radiant efficiency in the different ROIs over time after injection. Data are the median +/− interquartile range of 4 independent mice for Cy5-CRAMP-injected group.

Supplemental Figure 4

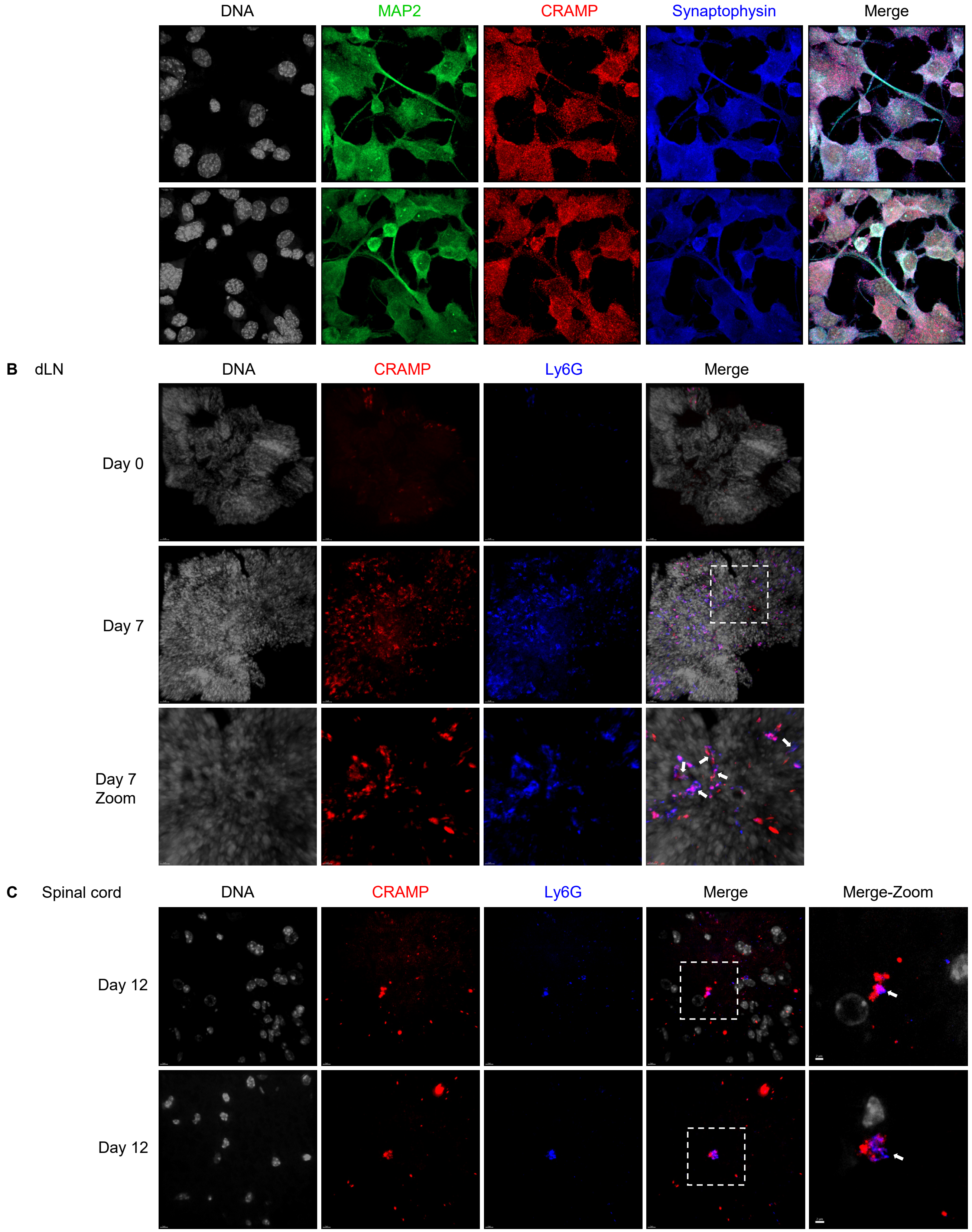

Figure S4 Cathelicidin expression by NSC-34 neurons and neutrophils in draining lymph nodes and spinal cord. (**A**) Confocal microscopy images of NSC-34 motor neuron-like cells stained for CRAMP (red), MAP2 (green), Synaptophysin (blue) and DNA (grey). Data are representative of 3 independent experiments. Original magnification, x63. Confocal microscopy images of draining lymph node (**B**) or spinal cord (**C**) section from WT mice immunized with MOG_{35-55}. Sections were stained for CRAMP (red), Ly6G (blue) and DNA (grey). Data are representative of 3 independent experiments. Arrows indicate neutrophil extracellular traps.

Supplemental Figure 5

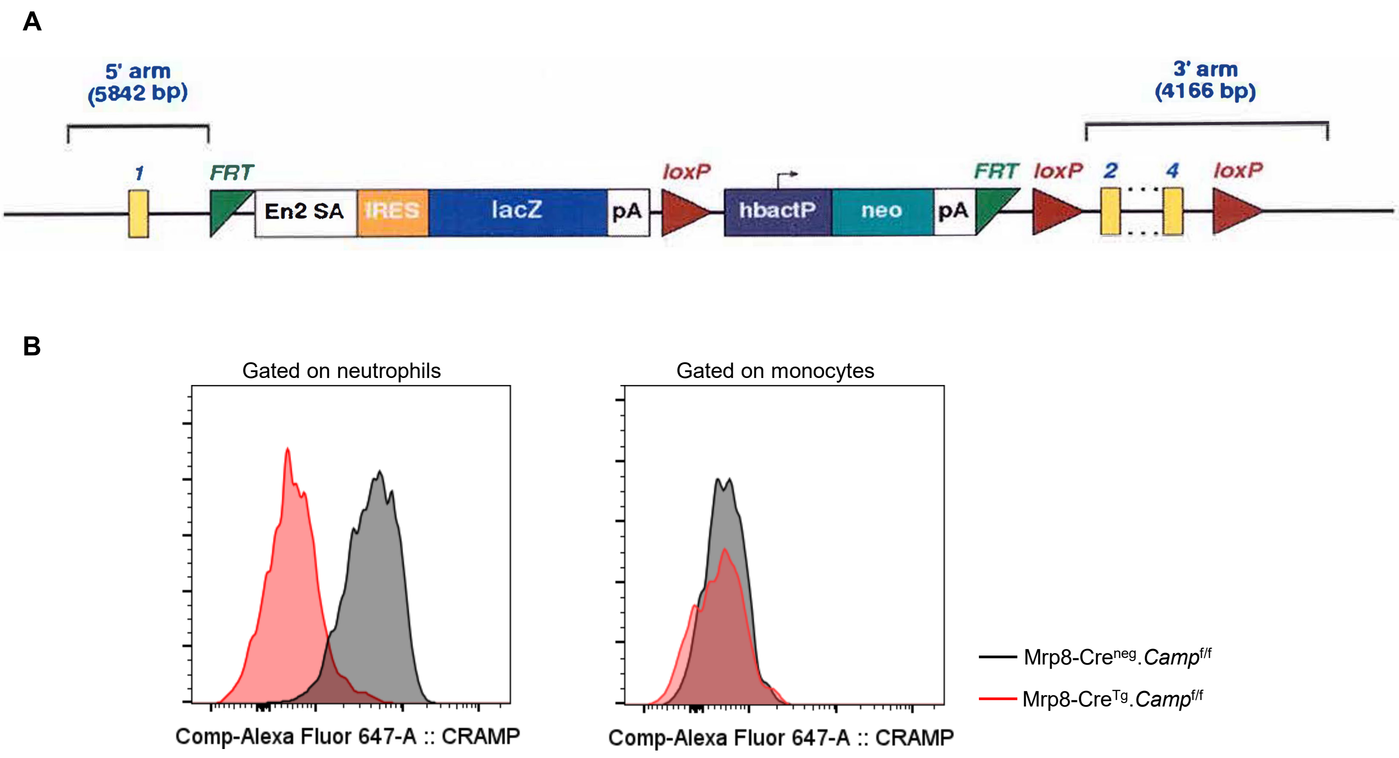

Figure S5 Generation and validation of the Mrp8-Cre^{Tg}.*Camp*$^{f/f}$ mice. (A) Representation of the *Camp*$^{tm1a(EUCOMM)HMGO}$ allele in ES cells (JM8A3.N1; cell clone ID HEPD0722_1_E10; MGl:4950203) targeting the *Camp* locus (from EUCOMM). **(B)** Flow cytometry analysis was performed from blood of Mrp8-Cre^{neg}.*Camp*$^{f/f}$ and Mrp8-Cre^{Tg}.*Camp*$^{f/f}$ mice. Cells were stained for CD45, CD11b and Ly6G to define neutrophils and Ly6C to define monocytes and then intracellularly stained for CRAMP. Data are representative of 3 independent experiments with 3 independent mice per group.

Supplemental Figure 6

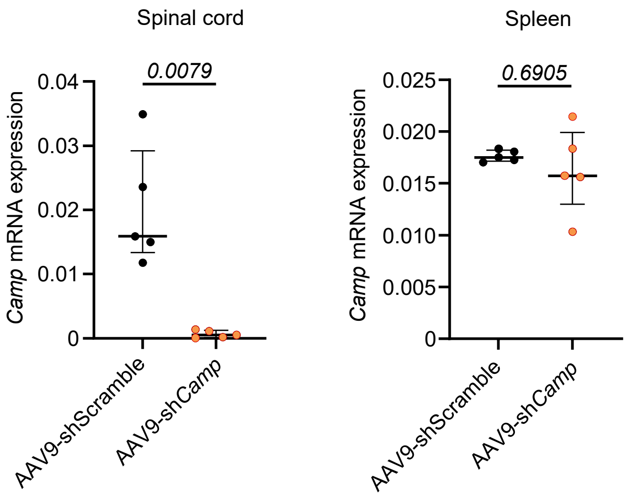

Figure S6 Specific knockdown of *Camp* expression in the spinal cord by intrathecal injection of AAV9-sh*Camp*. RT-qPCR analysis of spinal cord and spleen from C57BL/6 mice treated with i.t. injection of AAV9-sh*Camp* or AAV9-shScramble at day -7. Data are the median +/− interquartile range of 5 independent mice per group.

Supplemental Figure 7

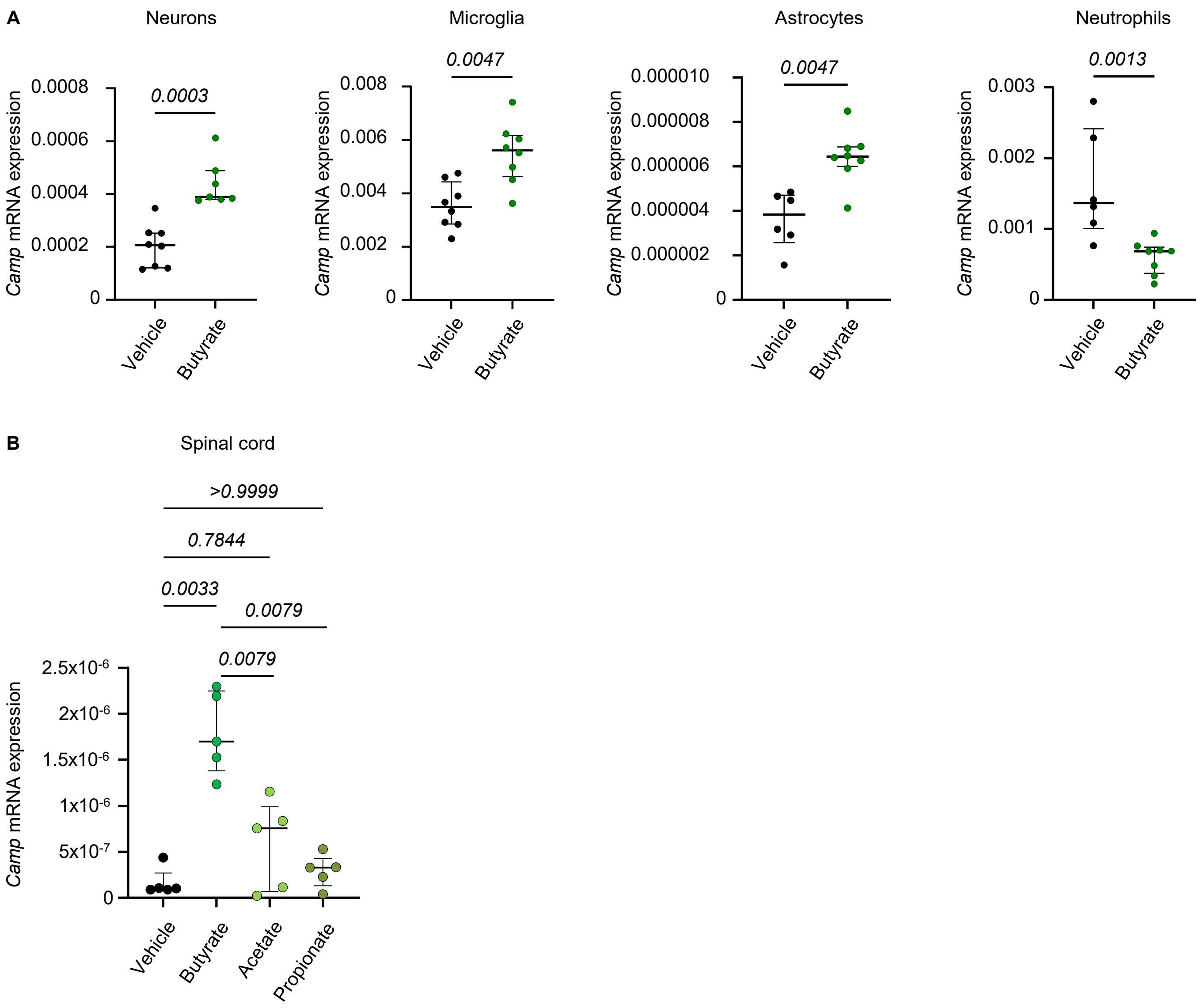

Figure S7 Short chain fatty acids stimulate *Camp* expression in the spinal cord. (**A**) WT mice were treated in drinking water with sodium butyrate (10 g.L^{-1}) for 7 days. Neurons, microglia, and astrocytes were isolated from CNS, and neutrophils from blood, by magnetic cell separation (Miltenyi), and *Camp* expression determined by RT-qPCR. Data are the median +/− interquartile range of 6 to 8 independent mice per group from 2 independent experiments. (**B**) WT mice were treated in drinking water with sodium butyrate, sodium acetate or sodium propionate (10 g.L^{-1}) for 7 days. Spinal cord was recovered, and *Camp* expression determined by RT-qPCR. Data are the median +/− interquartile range of 5 independent mice per group from 2 independent experiments.

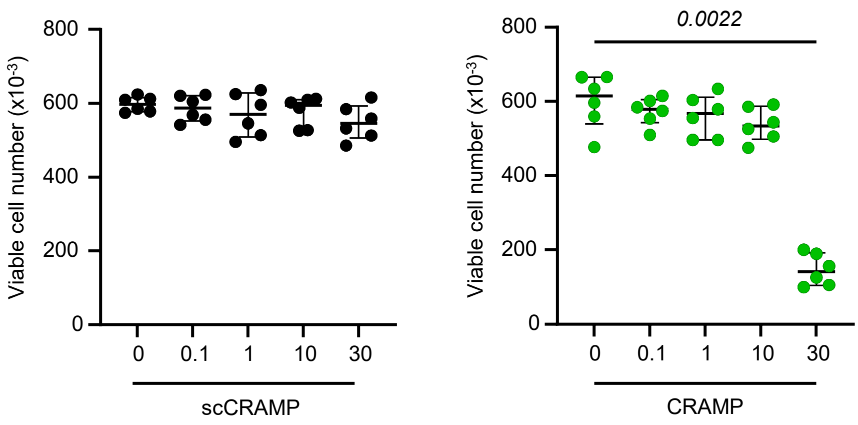

Figure S8 Toxicity of high dose of CRAMP on splenocyte culture. Splenocytes were recovered 10 days after EAE induction in WT mice and cultured for 3 days with MOG_{35-55} in pro-Th17 conditions with growing doses ($\mu g.mL^{-1}$) of scramble (sc)CRAMP or CRAMP. The number of viable cells was measured after trypan blue staining. Data are the median +/− interquartile range of 3 independent experiments.

Supplemental Figure 9

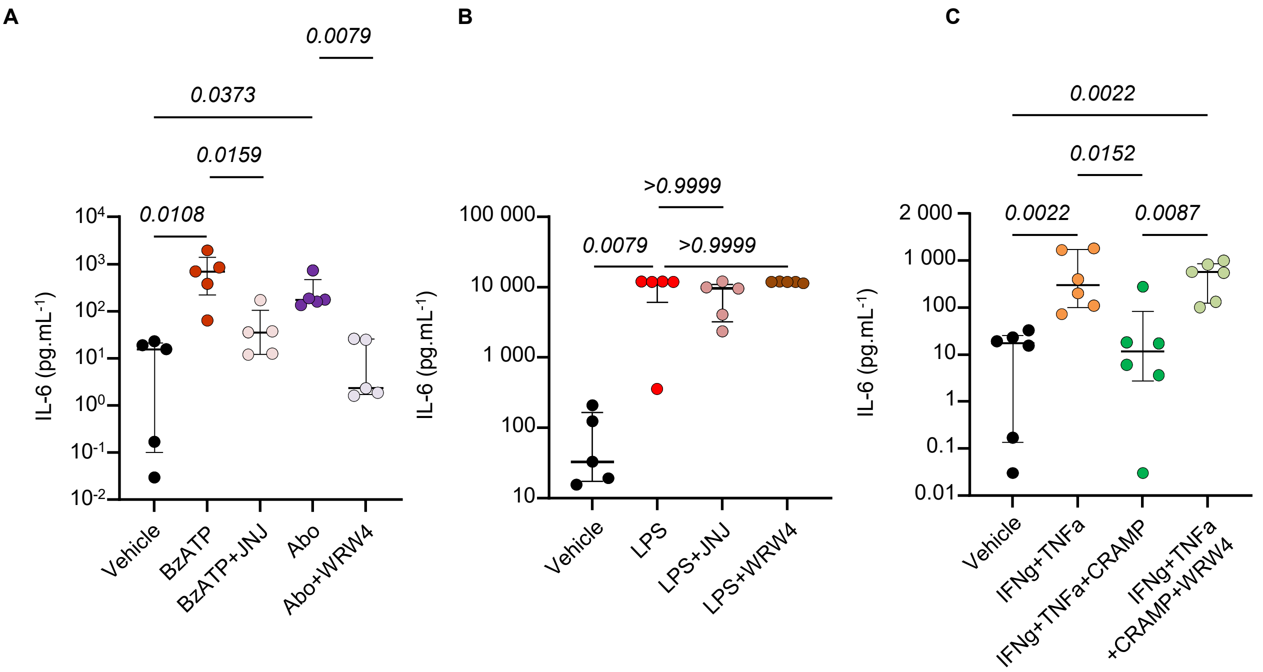

Figure S9 Impact of FPR2 and P2X7R antagonists on microglia activation. Microglia culture was prepared from C57BL/6 mice and activated or not with (**A**) FPR2 agonist (Amyloid-β_{1-42} oligomer (Abo), 10 µM), or P2X7R agonist (BzATP triethylammonium salt, 300 µM); (**B**) TLR4 agonist (LPS, 100 ng/mL); (**C**) IFNg+TNFa (50 ng.mL^{-1} and 20 ng.mL^{-1}, respectively). Cells were incubated for 24 h. Where indicated, FPR2 antagonist WRW4 or P2X7R antagonist JNJ-47695567 (JNJ) was added 2h before addition of the respective agonists; in C, CRAMP$_{1-39}$ (10 µg.mL^{-1}) was added 2 h after IFNg+TNFa. Cytokine levels were measured in the supernatant by multiplex ELISA. Data are the median +/− interquartile range of 5 to 6 independent wells from 2 independent experiments.

Supplemental Figure 10

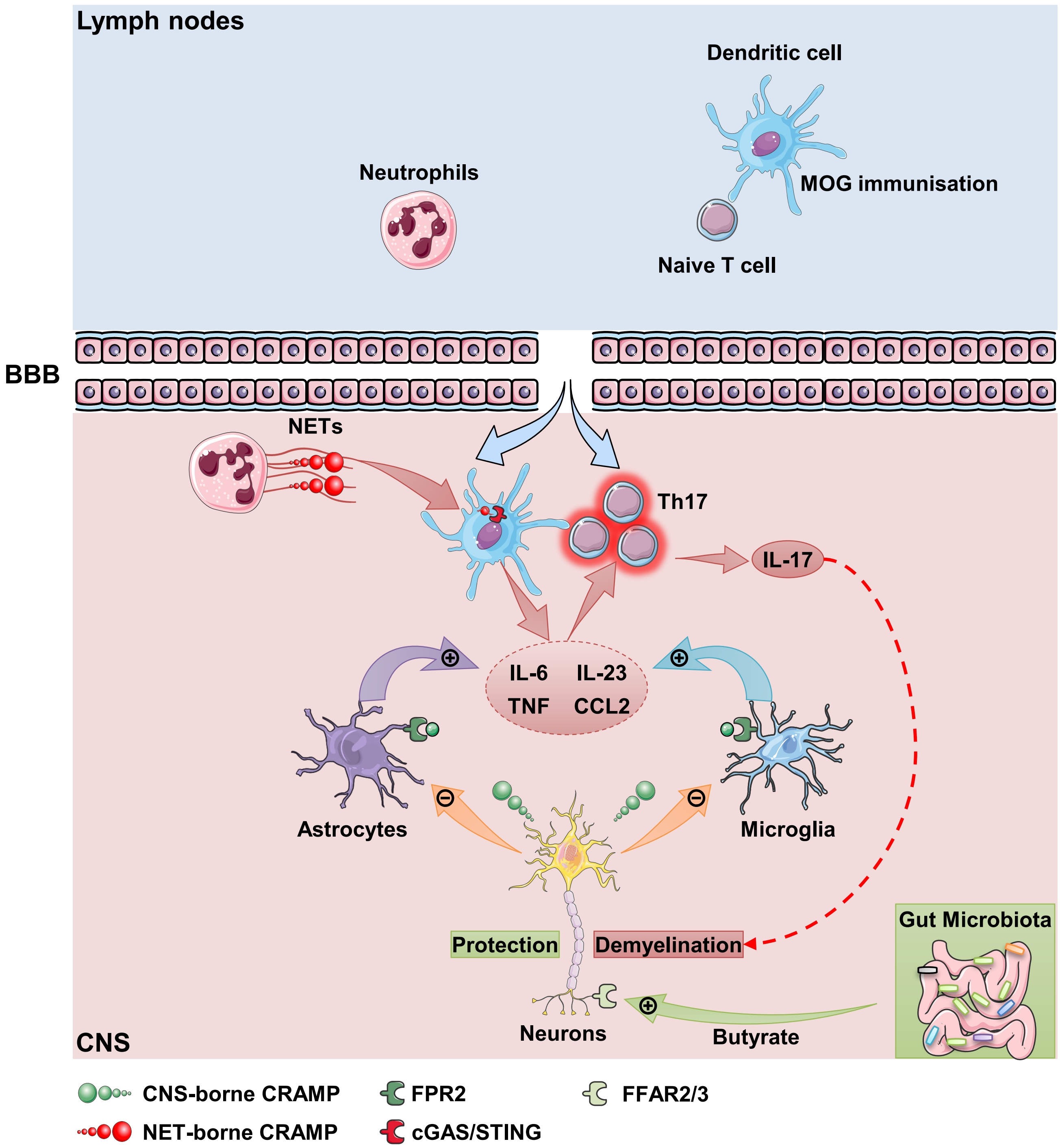

Figure S10 Cathelicidin antimicrobial peptide (CRAMP) exerts opposite roles in neuroinflammation depending on its cellular source. During experimental autoimmune encephalomyelitis (EAE), CRAMP is expressed locally in the CNS both by infiltrated neutrophils and by neural cells. CRAMP from neutrophil extracellular traps (NETs) is mandatory for the development of EAE by stimulating pro-Th17 cytokines by dendritic cells *via* the cGAS/STING pathway in the CNS. At the opposite, CRAMP from neurons shows protective function against EAE by inhibiting the activation of astrocytes and microglia through the FPR2 receptor expressed by both cell types. Expression of neuron-borne CRAMP can be stimulated via FFAR2/3 by butyrate, a gut microbiota-derived metabolite, providing a therapeutic opportunity to prevent EAE development.
